# Targeted proteome analysis of single-gene deletion strains of *Saccharomyces cerevisiae* lacking enzymes in the central carbon metabolism

**DOI:** 10.1371/journal.pone.0172742

**Published:** 2017-02-27

**Authors:** Fumio Matsuda, Syohei Kinoshita, Shunsuke Nishino, Atsumi Tomita, Hiroshi Shimizu

**Affiliations:** 1 Department of Bioinformatic Engineering, Graduate School of Information Science and Technology, Osaka University, Suita, Osaka, Japan; 2 RIKEN Center for Sustainable Resource Science, Tsurumi-ku, Yokohama, Japan; Universite Paris-Sud, FRANCE

## Abstract

Central carbon metabolism is controlled by modulating the protein abundance profiles of enzymes that maintain the essential systems in living organisms. In this study, metabolic adaptation mechanisms in the model organism *Saccharomyces cerevisiae* were investigated by direct determination of enzyme abundance levels in 30 wild type and mutant strains. We performed a targeted proteome analysis using *S*. *cerevisiae* strains that lack genes encoding the enzymes responsible for central carbon metabolism. Our analysis revealed that at least 30% of the observed variations in enzyme abundance levels could be explained by global regulatory mechanisms. A enzyme-enzyme co-abundance analysis revealed that the abundances of enzyme proteins involved in the trehalose metabolism and glycolysis changed in a coordinated manner under the control of the transcription factors for global regulation. The remaining variations were derived from local mechanisms such as a mutant-specific increase in the abundances of remote enzymes. The proteome data also suggested that, although the functional compensation of the deficient enzyme was attained by using more resources for protein biosynthesis, available resources for the biosynthesis of the enzymes responsible for central metabolism were not abundant in *S*. *cerevisiae* cells. These results showed that global and local regulation of enzyme abundance levels shape central carbon metabolism in *S*. *cerevisiae* by using a limited resource for protein biosynthesis.

## Introduction

Central carbon metabolism is an essential system of the living organisms. Active ethanol fermentation by the budding yeast, *Saccharomyces cerevisiae*, is a model of the eukaryotic central metabolism due to its importance in brewing industries, as well as its similarity to the Warburg effect in cancer cells [1]. It has been revealed that glycolytic flux is maintained via a complex control of metabolic reactions. For example, the allosteric regulation of phosphofructokinase (PFK) is important in glycolysis control [2]. A glycerol pathway has been considered as a drain for excess reducing power to avoid a redox imbalance inside the cells [3]. The post-translational regulation of enzymes also controls the central carbon metabolism [4–6].

Furthermore, it has been reported that the central carbon metabolism is controlled through a modulation of enzyme abundances in response to environmental conditions [7–11]. The regulation of enzyme abundance levels also works in mutant strains lacking genes of central carbon metabolism related enzymes. It is because metabolic imbalances caused by the gene deletions are compensated by increase and decrease in the abundance of other enzyme proteins to avoid a breakdown of metabolic systems [12]. The targeted proteome analysis showed that the expression of several glycolytic enzymes, including Pgi1, Tdh2, and Eno1 were increased in BY4742 pfk1Δ strain lacking *PFK1* gene, suggesting that the loss of the α subunit of PFK was counteracted by an activation of glycolysis [13]. The distant parts of the metabolic network were modulated to compensate the loss of a single enzyme protein since the expression of several TCA cycle related enzymes, such as Cit1, Kgd1, and Fum1, were increased in the BY4742 zwf1Δ strain [13]. The metabolic flux analysis also revealed that knockout of glucose-6-phosphate dehydrogenase gene (*ZWF1*) caused an up-regulation of TCA cycle flux level [14].

The protein abundance profiles is likely regulated and constrained by several factors. First, the regulation of enzyme abundance is constrained by the cost of amino acid for protein biosynthesis [15, 16]. Given that glycolytic enzymes such as Tdh3 are one of the most abundant proteins in *S*. *cerevisiae*, the increase and decrease in these abundance should largely affect the composition and resource allocation of the *S*. *cerevisiae* proteome [17]. Second, transcriptome analyses indicated coordinated expressions of genes encoding enzymes [18–20]. For instance, a number of genes encoding glycolytic enzymes, such as *TDH3* and *TPI1*, form a co-expression module through the regulation of transcriptional activators, such as Gcr1 and Gcr2 [18, 21]. Third, central carbon metabolism could be controlled by local regulation of a small number of enzyme genes. For instance, the promoter activity of a minor isoform of the pyruvate decarboxylase gene (*PDC5*) is specifically increased in a mutant strain lacking the major isoform gene (*PDC1*) to functionally compensate the reduced pyruvate decarboxylase activity [22]. Enzyme abundance levels, however, do not always correlate with their gene expression levels [19, 23, 24], and the relative contribution of these regulation to the maintenance of central carbon metabolism remains unclear.

In this study, we have addressed this issue by the direct determination of enzyme abundance in the single gene-deletion mutant strains of *S*. *cerevisiae* by targeted proteomics using liquid chromatography-tandem quadrupole mass spectrometry (LC-MS/MS). The selected reaction monitoring (SRM) assay for the central metabolism related enzymes of *S*. *cerevisiae* has been extensively developed by excellent efforts of the ETH group [25–27]. The analysis of the enzyme abundance dataset revealed that the central carbon metabolism in *S*. *cerevisiae* is controlled through specific and coordinated regulations of enzyme abundance levels by using a limited resource for protein biosynthesis.

## Results and discussion

### Targeted proteome analysis of single gene deletion mutants of *S*. *cerevisiae*

**Fig 1:**
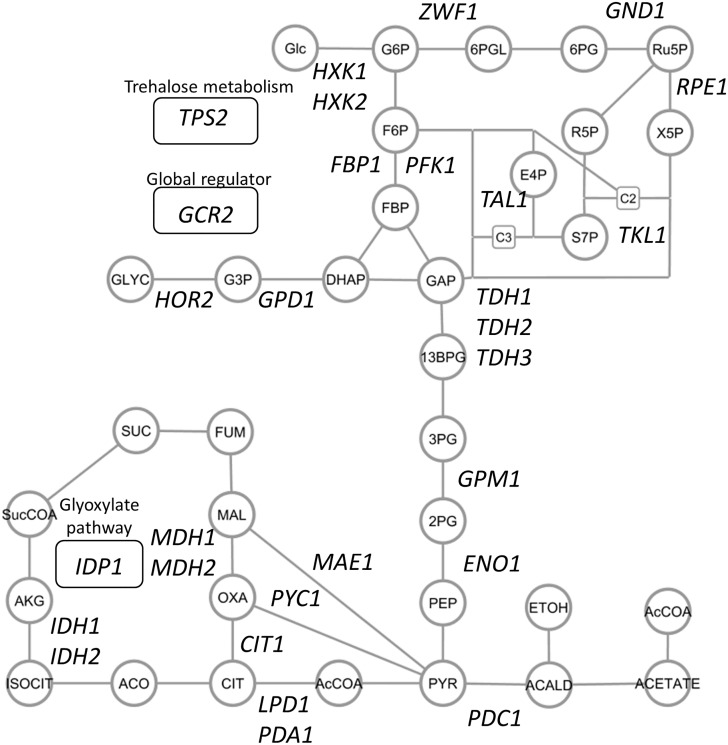
Central metabolism related genes whose single-gene deletion mutants were investigated in this study. Deleted genes were shown in the simplified metabolite pathways. Circles and lines represent metabolites and reactions, respectively. Genes related to the trehalose metabolism, glyoxylate pathway, and the global regulation were shown in round rectangles. Abbreviations: Glc: glucose, G6P: glucose 6-phosphate, F6P: fructose 6-phosphate, FBP: fructose 1,6-bisphosphate,DHAP: dihydroxyacetone phosphate, GAP: glyceraldehyde 3-phosphate, 13BPD: 1,3-bisphosphoglycerate, 3PG: 3-phosphoglycerate, 2PG: 2-phosphoglycerate, PEP: phosphoenolpyruvate, Pyr: pyruvate, AcCoA: acetyl-coenzyme A, 6PGL: 6-phosphogluconolactone, 6PG: 6-phosphogluconate, Ru5P: ribulose 5-phosphate, R5P: ribose 5-phosphate, Xu5P: xylulose 5-phosphate, S7P: sedoheptulose 7-phosphate, E4P: erythrose 4-phosphate, ACALD: acetoaldehyde, ETOH: ethanol, CIT: citrate, ISOCIT: isocitrate, ACO: anonitate, AKG: alpha-ketoglutarate, SucCOA: succinyl coenzyme A, SUC: succinate, FUM: fumarate, MAL: malate, OXA: oxaloacetate, G3P: glycerol-3-phosphate, GLYC: glycerol.

Protein abundance profiles of the central metabolism-related enzymes were obtained from the wild type (BY4742), 29 single-gene deletion strains lacking enzyme genes responsible for the central carbon metabolism and a gene encoding transcriptional activator (Fig 1 and S1 Table). The single-gene deletion strains were arbitrarily selected based on the growth phenotype in the Saccharomyces genome database (SGD, http://www.yeastgenome.org/). The single-gene deletion strains were used without checking these genotype in detail [28]. These strains were batch cultivated in shake flasks with triplicate, from which crude protein mixtures were extracted at an exponential growth phase. The trypsin-digested peptides were analyzed by the SRM assay methods (S2 Table). In order to compare protein levels accurately, fully ^13^C-labeled peptide samples were prepared from yeast strain S288C cells grown in SD medium containing [U-^13^C] glucose as the sole carbon source. The fully ^13^C-labeled peptide samples were used as the internal standards for all analyses in this study.

**Fig 2:**
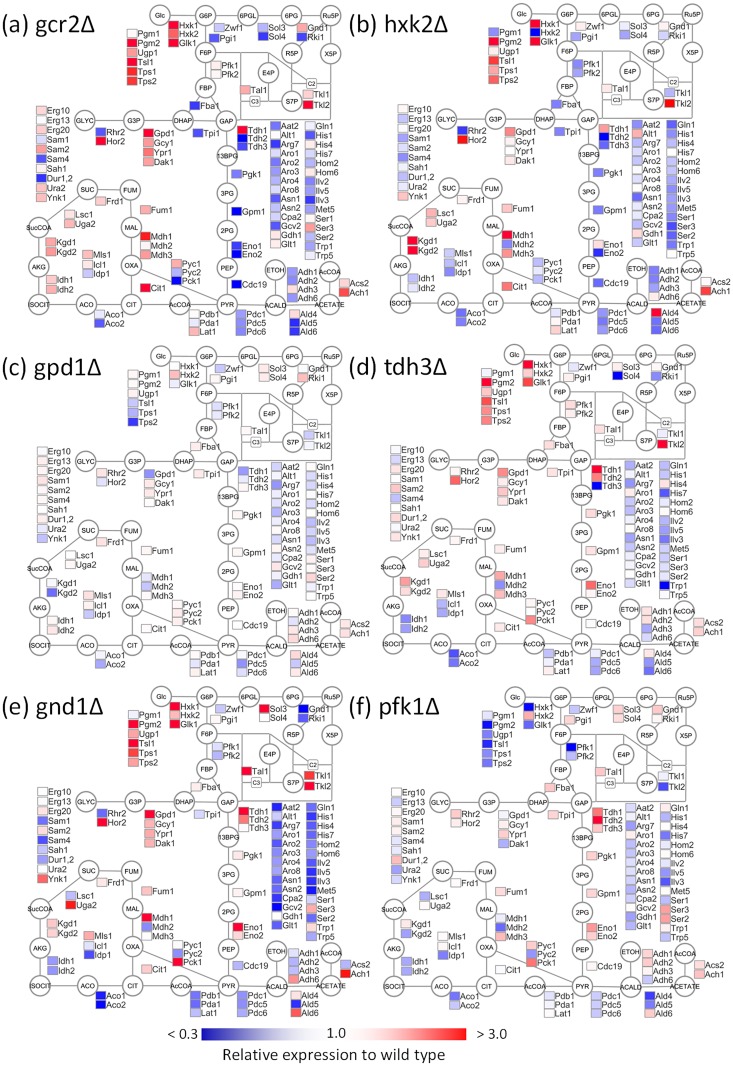
Protein abundance profiles of the central metabolism related enzymes in *Saccharomyces cerevisiae*. The data obtained from (a) gcr2Δ, (b) hxk2Δ, (c) gpd1Δ, (d) tdh3Δ, (e) gnd1Δ, and (f) pfk1Δ strains lacking *GCR2*, *HXK2*, *GPD1*, *TDH3*, *GND1*, and *PFK1* genes were represented, respectively. Enzyme abundance levels relative to that of wild type (BY4742) were shown as a heat map of simplified metabolite pathways. All abbreviations were described in the legend for Fig 1.

The SRM assays successfully determined the levels of peptides derived from the 110 enzymes (S3 Table and S1 Fig). For example, a comparison of the protein abundance profiles of the wild type (BY4742) with those of the mutant strains lacking a transcriptional activator of glycolysis, Gcr2 (denoted as gcr2Δ, Fig 2a) and a major isoform of hexokinase, Hxk2 (hxk2Δ, Fig 2b) showed significant changes in abundances of 37 and 27 enzymes, respectively (two-side Welch’s t-test, α = 0.01, S1 Table). The pattern of enzymes abundance in gcr2Δ strain was essentially similar to the literature reported transcriptome data of glycolytic genes [21]. The large perturbation of the enzyme abundance profile caused a decrease in the specific growth rate of gcr2Δ strain (73% of the wild type and S1 Table). Furthermore, the levels of trehalose metabolism-related enzymes such as Pgm2, Ugp1, Tps1, Tps2, and hexokinases, such as Hxk1 and Glk1, commonly increased in both strains [21]. On the other hand, the abundance levels of glycolytic enzymes, such as Fba1, Tpi1, and Cdc19, decreased. These results suggested that the single deletions of central metabolism-related enzyme genes such as Hxk2 caused an expressional modulation of numbers of enzymes.

### Comparison with specific growth rates

**Fig 3:**
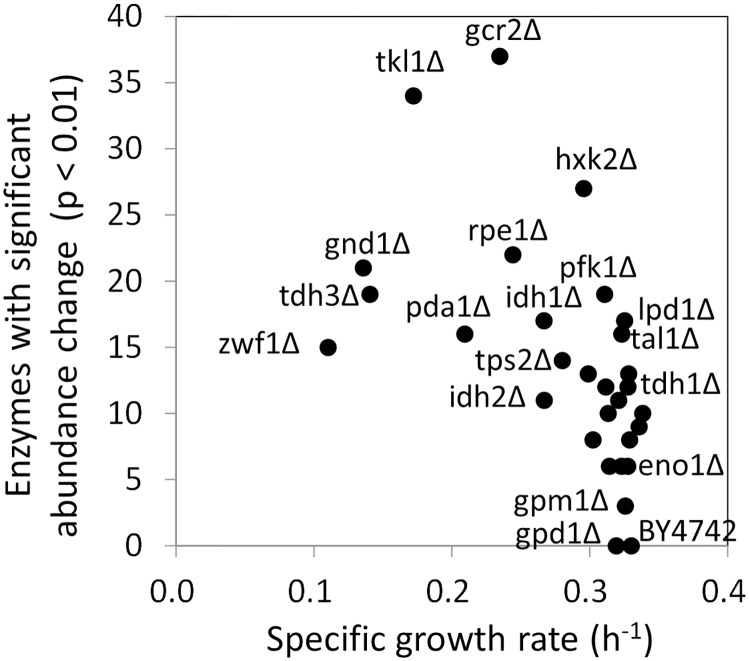
Relationship between specific growth rates and numbers of enzymes whose expression levels were significantly changed. The specific growth rates were determined from the flask-scale batch cultivation data.

The specific growth rates of each strain were compared with the number of significantly modulated enzymes (S1 Table and Fig 3). The scatter plot (Fig 3) showed that there was a negative relationship between the specific growth rates and the number of significantly modulated enzymes. While the mutant strains with smaller number of significantly modulated enzymes showed firster specific growth rates such as in BY4742 and gpd1Δ, the mutant strains with slower growth rates tended to show larger modulation in the enzyme abundance.

For instance, the gpd1Δ strain lacking an isoform of glycerol-3-phosphate dehydrogenase, Gpd1, showed a similar specific growth rate to that of wild type, since level of no enzyme was significantly modulated (two-side Welch’s t-test, α = 0.01) (Figs 3 and 2c). The results also indicated that Gpd1 plays a minor role in the culture conditions since it has been reported that Gpd2 plays the major role under anaerobic conditions, and the deletion of *GPD2* reduced growth under anaerobiosis [29–31]. In contrast, a slow growth phenotype as well as a large perturbation in the enzyme abundance profile was observed with deletion mutants, such as those lacking oxidative pentose phosphate pathway (gnd1Δ, tkl1Δ, zwf1Δ, and rpe1Δ) and glycolysis (tdh3Δ and pda1Δ) genes. For instance, tdh3Δ strain lacking a major isoform of glyceraldehyde 3-phosphate dehydrogenase, Tdh3, showed a 58% decrease in the specific growth rate (Fig 3) and a large perturbation in the enzyme abundance profile (Fig 2d). Moreover, the abundances of 21 enzymes significantly changed in gnd1Δ strain lacking a major isoform of 6-phosphoglucolate dehydrogenase, Gnd1 (Fig 2e).

**Fig 4:**
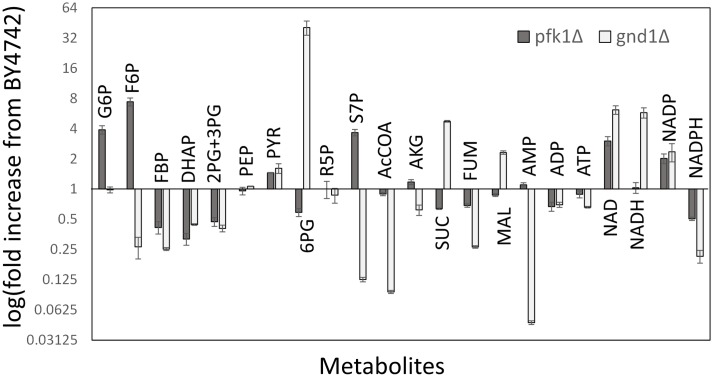
Metabolite profiles in pfk1Δ and gnd1Δ strains. Fold increase with respect to the BY4742 strain is shown for each metabolite. Data are shown as the means of triplicate analyses with standard deviations. All abbreviations were described in the legend for Fig 1.

However, the negative relationship was not the case for lpd1Δ, hxk2Δ, and pfk1Δ because the mutants showed a large perturbation in the enzyme abundance profile and similar specific growth rates to that of wild type (Fig 3). The results suggested that the loss of deleted gene functions could be backed up or compensated by the modulation of abundance of a number of enzymes. For example, while the abundances of 27 enzymes significantly changed in hxk2Δ (Fig 2b), the specific growth rate was 90% of that of wild type (Fig 3). In the case of pfk1Δ strain lacking α subunit of phosphofruktokinase, Pfk1, significant variations were observed for 19 enzymes in the enzyme abundance profile data (Fig 2f). In order to investigate a relationship between the enzyme abundance profile and cell metabolism, a metabolic profiling analysis was conducted for BY4742 and pfk1Δ strains (Fig 4). The metabolic profile data showed that the loss of Pfk1 caused an accumulation of up-stream metabolites, including fructose-6-phosphate (F6P) and glucose-6-phosphate (G6P), as well as a decrease in down-stream metabolites such as fructose-1,6-bisphosphate (FBP) and dihydroxyacetone phosphate (DHAP). The level of sedoheptulose-7-phosphate (S7P) was also drastically increased in pfk1Δ strain by unknown reasons. However, since the metabolite levels in lower part of glycolysis were similar to those of the wild type, the effect of Pfk1 deletion on the metabolism was likely to be restricted in the upper part of the glycolysis by the modulation of expression of 19 enzymes. These results suggested that the single deletions of central metabolism-related genes caused a modulation of numbers of enzyme abundances to maintain the metabolic homeostasis in deleted gene dependent manners.

### Resource allocation for enzyme biosynthesis

**Fig 5:**
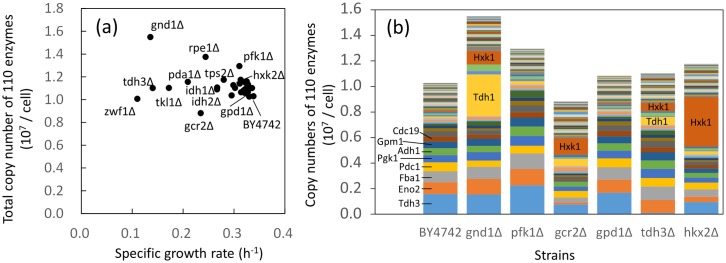
The composition of enzyme proteins. (a) Relationship between specific growth rates and total copy numbers of the 110 enzymes investigated in this study. (b) Composition of the 110 enzymes in selected strains. In this study, the copy number indicates a molecular number of expressed protein. All data were calculated from the dataset by Kulak *et al* [17] and the targeted proteome data obtained in this study. Data of other strains are shown in S2 Fig. Data are shown as means of triplicate analyses. Strain and enzyme names were represented in the Figures.

The cost of protein investment in cellular metabolic function can be estimated from the absolute protein abundance data [32]. The absolute protein abundance or copy numbers of expressed proteins per cell have been determined comprehensively for the wild type *S*. *cerevisiae* strains in previous studies [17, 33–35]. Here, the copy number means a molecular number of expressed protein in this study. For instance, the dataset by Kulak *et al* showed that the total copy number of the 110 enzymes analyzed in this study was 1.03 × 10^7^ copies/cell [17]. The dataset also indicated that glycolysis related enzymes such as Tdh3 are among the most abundantly expressed of the 110 enzymes. An essentially similar trend was also observed in other datasets [33–35]. Here, using the dataset by Kulak *et al* as the copy numbers of the 110 enzymes in the wild type strain, the copy numbers in each mutant strain were calculated from the targeted proteome data obtained in this study (Fig 5 and S2 Fig).

The estimated total copy numbers of the 110 enzymes were compared with the specific growth rates (Fig 5a). The result showed that the total copy numbers increased exceptionally in several strains, such as gnd1Δ strain lacking the gene encoding glucose-6-phosphate dehydrogenase (1.55 × 10^7^ copies/cell) and the rpe1Δ strain lacking the gene encoding ribulose-5-phosphate 3-epimerase (1.38 × 10^7^ copies/cell) (Fig 5a). The composition of the protein abundance profile showed that the copy numbers of Tdh1 and Hxk1 drastically increased in gnd1Δ (Fig 5b). The copy numbers of Tdh1, Hxk1, and Tdh3 also increased in rpe1Δ (Fig 5b). The increase and decrease in the abundance of other enzymes, such as those involved in amino acid biosynthesis and the TCA cycle, had little effect on the copy number profiles, since the copy numbers of these enzymes in the wild type were significantly lower than those of glycolytic enzymes. In gnd1Δ and rpe1Δ, the specific growth rates were reduced partly because a large resource was additionally allocated for protein biosynthesis in response to serious metabolic perturbations caused by deletion of *GND1* and *RPE1*. The comparison between the total copy numbers and the specific growth rate also indicated that gcr2Δ strain contained smaller copy numbers of the 110 enzymes than the wild type strain did (Fig 5a). Although the abundance of Hxk1 showed an increase, the abundances of other glycolytic enzymes decreased uniformly due to loss of the global regulator of glycolysis (Gcr2) as mentioned earlier (Fig 5b).

In the case of other mutant strains, the comparison between the total copy numbers and the specific growth rates (Fig 5a) showed that the total copy numbers of the 110 enzymes tended to increase but less than 115% of that of the wild type (Fig 5a). For example, the total copy numbers in gpd1Δ, tdh3Δ, and hxk2Δ were 105, 107, and 114% of that of the wild type, respectively (Fig 5a). Similar trends were observed for the numbers of amino acids required for the biosynthesis of the 110 enzymes (S2 Fig) as well as from the analyses using datasets produced by other researches (S3 Fig) [33, 35]. The results suggested that functional compensation of the deleted enzyme was attained by using more resources for protein biosynthesis under a constraint in the available resources for the synthesis of glycolytic enzymes.

The presence of a constraint was also evident from the enzyme compositions (Fig 5b). For example, Tdh3, a major isoform of glyceraldehyde-3-phosphate dehydrogenase, was one of the most abundant enzyme in BY4742 (Fig 5b). However, the total copy numbers of 110 enzymes in the tdh3Δ strain increased to 107% of that of the wild type, since the copy numbers of Tdh1, Hxk1, and other major glycolytic enzymes increased evenly in tdh3Δ (Fig 5b). Furthermore, in the hxk2Δ strain, the copy number of Hxk1 drastically increased for functional compensation of the isozyme (Fig 5b). The specific up-regulation of Hxk1 expression in hxk2Δ seemed reasonable as a backup for Hxk2 deletion, since the compensatory expression of *HXK1* and *HXK2* was observed under conditions having different carbon sources [36]. Genetic analysis also found that Hxk2 functions in the nucleus to repress *HXK1* and *GLK1* expression [37]. However, the total copy number was kept at 114% of the wild type, because the expressions of other enzymes were uniformly reduced in hxk2Δ (Fig 5b).

### Enzyme-enzyme co-abundance network uncovered coordinated regulations of enzyme levels

**Fig 6:**
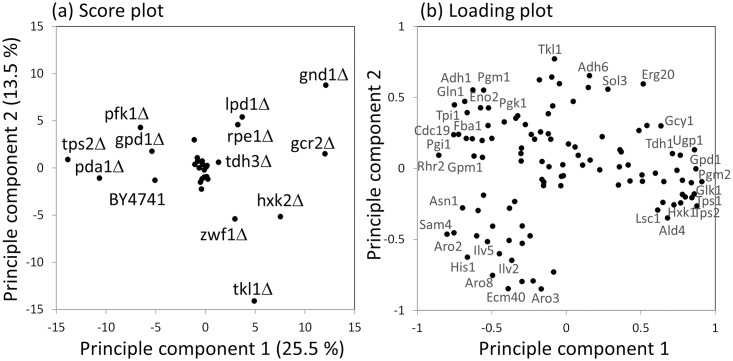
Principle Component Analysis (PCA) of the enzyme abundance data. (a) PCA score plot for mutants. The mean, log2-transformed and z-scored enzyme abundance data was used for the analysis. The score plots of principle component 1 and 2 are shown in the figure. (b) Loading plot of principle component 1 and 2. Strain and enzyme names were represented in the Figures.

Effects of gene deletions on the enzyme abundance profiles were compared by the principal component analysis (PCA). The result showed that a cluster of mutant strains was observed in the center of the PCA score plot (Fig 6a). Since the wild type (BY4742) was outside the cluster, a common change in enzyme abundance was shared among the strains in the cluster. The enzyme expression profile data indicated that the abundances of enzymes, such as Hxk1, Pgm2, and Tps2 were commonly increased in these strains (Fig 6b and S1 Fig). The score plot also suggested that the enzyme abundance profiles were significantly changed in the deletion strains of genes related to pentose phosphate pathway (gnd1Δ, zwf1Δ, tkl1Δ, and rpe1Δ), entry point of glycolysis (hxk2Δ and pfk1Δ), pyruvate dehydrogenase complex (pda1Δ, lpd1Δ), trehalose biosynthesis (tps1Δ and tps2Δ), and gcr2Δ (Fig 5a). Although these strains failed to form clusters in the score plot of PCA, coordinated regulations somehow work in these mutant strains, since the first and second principal components explained the 30.5% of total variations in the enzyme expression profiles (Fig 6a).

**Fig 7:**
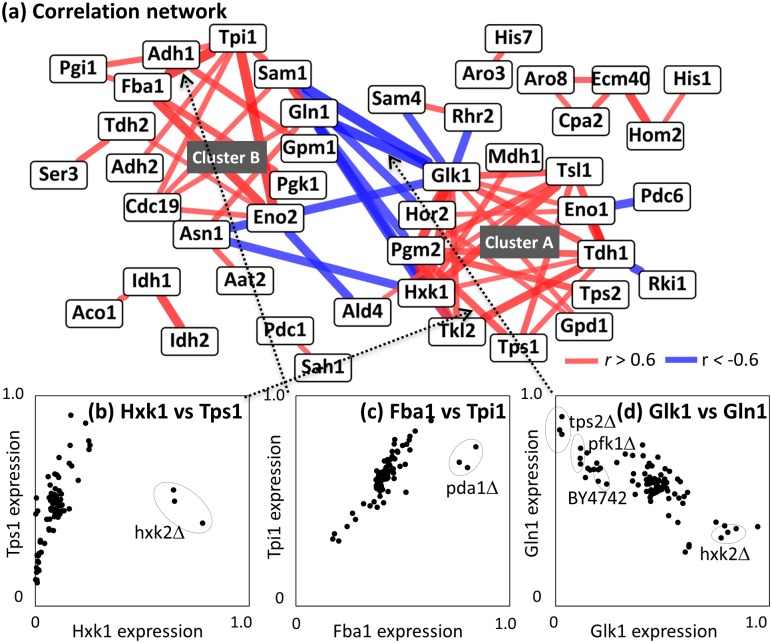
Enzyme-enzyme co-abundance analysis. (a) Enzyme-enzyme co-abundance network. Nodes and edges indicate enzymes and their correlations. Positive (r > 0.6) and negative (r < -0.6) correlations were indicated as red and blue, respectively. (b-d) Scatter plot between two enzyme expression data including (b) Hxk1 vs. Tps1, (c) Fba1 and Tpi1, and (d) Glk1 vs. Gln1. Dotted arrows indicate corresponding edges between two enzymes. Dotted circles represent data derived from specific mutants.

Coordination behind the regulation was investigated by construction of a co- abundance network of enzyme-enzyme pairs (Fig 7a). In this section, Spearman's rank-order correlation coefficients (*r*) were calculated among enzymes. In order to investigate effects derived from the deletion of other enzyme genes, the analysis was performed without using the data obtained from gcr2Δ and mutant strains of the protein of interest. For example, data obtained from gcr2Δ, hxk1Δ, and tps1Δ strains was omitted in the determination of correlation coefficients (*r*) between Hxk1 (a minor isoform of hexokinase) and Tps1 (a subunit of trehalose-6-phosphate synthase/phosphatase complex). It was because *GCR2* did not encode enzyme and the abundances of Hxk1 in hxk1Δ and Tps1 in tps1Δ did not reflect effects derived from the deletion of other enzyme genes. The results showed that a linear correlation was observed between Hxk1 and Tps1 abundances, whose correlation coefficient was determined to be 0.61 (Fig 7b). The correlation coefficients of Fba1/Tpi1 and Glk1/Gln1 pairs were also calculated to be 0.79 and -0.67, respectively (Fig 7c and 7d).

The positive and negative correlations were derived from coordinated regulations of enzyme abundance levels, since the distribution of correlation coefficients of all enzyme pairs is wider than null distribution (S4 Fig). The coordinated regulation of the abundance levels of enzyme proteins seems reasonable for controlling the glycolysis flux because an over-expression of the single enzyme failed to affect cell metabolism [38].

### Coordinated regulations by global regulators

**Fig 8:**
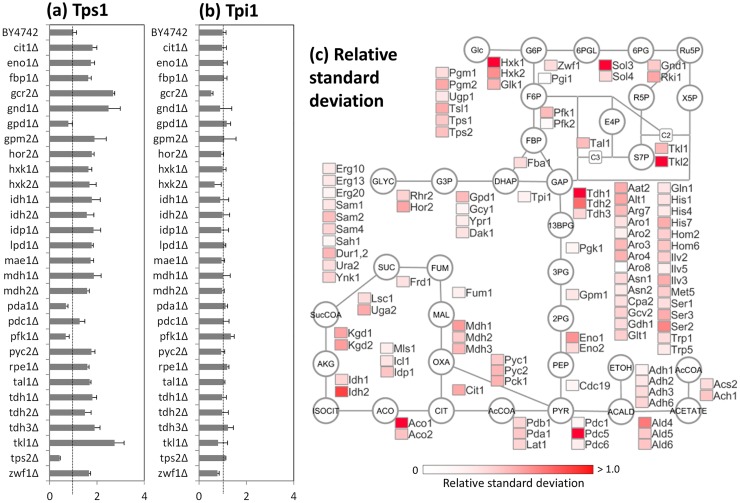
Variation of enzyme abundances among *S*. *cerevisiae* mutants. (a and b) Abundances of (a) Tps1 and (b) Tpi1 proteins in single gene deletion mutants. Lines indicate abundances of wild type (BY4742). Data are shown as the mean of triplicate analysis with standard deviations. (c) Relative standard deviations of enzyme expression levels among 30 wild type strain and mutants. The data were represented as a heat map in the simplified metabolic pathways.

The co-abundance network contains two large and several small clusters of positively correlated enzymes. The largest cluster (Cluster A in Fig 7a) contains enzymes responsible for the trehalose and glycerol metabolism, such as Tps1, Pgm2, Tsl1, and Gpd1, in addition to the glycolytic enzymes, including Tdh1, Eno1, and Hxk1. The coordinated regulation of enzyme abundance levels seemed to play a role in the metabolic adaptation, since the expression levels of Tps1 (a subunit of trehalose-6-phosphate synthase/phosphatase complex) were significantly increased in 14 single deletion mutants, including gcr2Δ and hxk2Δ (Fig 2a and 2b) with large variations among 30 strains (0.48–2.68 fold of the wild type strain, Fig 8a). A relative standard deviation (RSD) of Tps1 abundance was 0.58. The large variations in the expression levels were also observed for other enzymes in the cluster A (Fig 8c).

A comparison with the other proteomics data suggested that the enzymes in Cluster A is involved in the cellular response to osmotic stress under regulation of transcriptional activators (Msn2/4) mediating a general response to multiple stresses [8, 39, 40]. The results suggested that the coordinated regulation of enzyme abundance levels observed in the single gene deletion mutants would be connected with the stress response mechanisms in *S*. *cerevisiae*. It has also been reported that Msn2/4 controls the expression of all enzyme-encoding genes in the pathways for trehalose biosynthesis from glucose-6-phosphate (G6P) and its degradation to glucose [18, 39, 41]. The trehalose biosynthesis and degradation pathways form a metabolic shunt or a futile cycle consuming ATP. While trehalose itself protects subcellular structures against osmotic and other stresses [42, 43], it has been recently reported that the futile cycling of the trehalose shunt has a role in the regulation of glycolysis stability [44, 45]. The reports showed that the trehalose shunt is a mechanism to reduce the probability of unbalanced state glycolysis, because the glycolysis in *S*. *cerevisiae* is intrinsically unstable. The larger variation in the abundance of the trehalose metabolism related enzymes observed in this study suggested that the regulation is an important mechanism for metabolic adaptation of *S*. *cerevisiae* (Fig 8c).

Second largest cluster (Cluster B) includes several glycolytic enzymes, such as Cdc19, Tpi1, Eno2, Fba1, and Pgi1 (Fig 7a). Amino acid biosynthesis related enzymes, such as Gln1 and Asn1, were also included in this module. In contrast to the cluster A, these enzymes showed small variations in the abundance levels. For instance, the variation of triose-phosphate isomerase (Tpi1) abundance was within 0.51–1.34 fold of the wild type strain (Fig 8b), whose RSD level (0.13) was one of the lowest levels among all enzymes (Fig 8c). The fine-tuning occurred in the single gene deletion strains with significantly perturbed enzyme abundance profiles, because Tpi1 abundances were decreased in hxk2Δ, tkl1Δ, and zwf1Δ strains, while increased in pfk1Δ and tdh3Δ strains (Figs 2b, 2d, 2f and 8b).

The enzymes in the cluster B was partly coincidence with the enzymes whose abundances were decreased in the gcr2Δ strains (Figs 7a and 2a). These results suggested a fine-tuning of these enzyme abundance levels are under the control of Gcr1/2 transcription factor [21, 46–48]. Furthermore, the tight co-expression of enzymes might reflect additional functions of enzymes, such as a glycolytic metabolon complex using filamentous actin for stabilization [49].

In addition to the two large clusters, small clusters containing enzymes related to TCA cycle (Aco1, Idh1, and Idh2) and amino acids biosynthesis (Cpa1, Ecm40, Aro8, Hom2, and His1) were also found in the co-abundance network (Fig 7a). The retrograde control by RTG genes [50] and the general amino acid control by *GCN4* [51] seem to have some role behind the coordinated regulation.

The co-abundance network also showed that there were negative correlations between the enzymes in cluster A and B, such as the Glk1 (glucokinase)/Gln1 (glutamine synthetase) pair (*r* = -0.67, Fig 7d). These results suggested that there was an exclusive trend between the abundance of enzymes in the clusters. Although detailed mechanism remains unclear, the loading plot of PCA showed that the enzymes in cluster A (such as Tdh1, Tps1, and Glk1) and B (such as Tpi1, Cdc19, and Gln1) were plotted at the positive and negative ends of the principle component 1 (Fig 6b). Moreover, the enzyme abundance profile of hxk2Δ and gnd1Δ showed an increased abundance of Glk1 and the trehalose metabolism related enzymes as well as a decreased abundance of Gln1 and the glycolysis related enzymes (Figs 2b, 2e and 7d). Opposite profiles (increased and decreased abundances of Gln1 and Glk1, respectively) were observed for pfk1Δ and tps2Δ strains (Figs 2f and 7d).

### Mutant-specific regulation of enzyme expression levels

The scatter plot of the Hxk1 and Tps1 abundances showed that the data obtained from the hxk2Δ were outliers (Fig 7b). The specific increase in Hxk1 abundance in hxk2Δ seemed reasonable, as mentioned previously. However, roles of expression modulation of other distant enzymes shown in hxk2Δ, such as the up-regulation of two subunits of α-ketoglutarate dehydrogenase (Kgd1 and Kgd2) in the TCA cycle, were unclear (Fig 2b). Since the abundances of Kgd1 and Kgd2 in the hxk2Δ were not only 3.3 and 3.0 times larger than that of BY4742 but also the most abundant among the strains examined in this study. It has been reported that the hxk2-null mutant strain showed a reduction of the glucose repression and fully oxidative growth at high glucose concentrations [52]. The increase in the abundance of Kgd1 and Kgd2 proteins should be responsible for the oxidative metabolism. The targeted proteome data indicated that the regulation mechanism is likely to be specifically working in the hxk2Δ strain.

**Fig 9:**
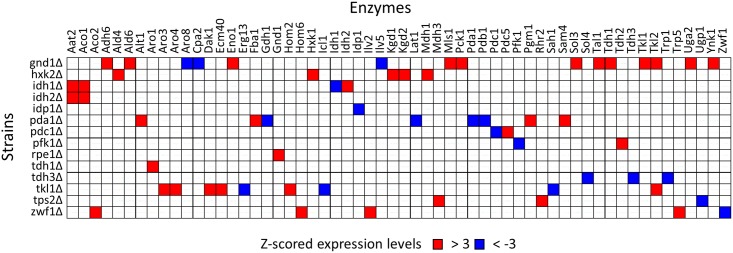
Mutant-specific regulation of enzyme abundance levels. Extreme increase and decrease in enzymes abundance among single gene deletion mutants. Colored cells indicate enzymes for which the z-scored abundance levels were over 3 (red) and below -3 (blue) in this strain. The blank strains and enzymes with no extreme increase and decrease are not shown in the Figure.

Similar mutant-specific and extreme modulations were investigated in the enzyme abundance profile data. Here, the variations in abundances of each enzyme among the 30 strains were normalized to z-scores. For example, the z-scored levels of Hxk1 and Kgd1 in hxk2Δ were 4.7 and 3.7, respectively, indicating that the abundance of Hxk1 and Kgd1 increased extraordinarily in hxk2Δ, among the 30 strains (Fig 9). The analysis of the whole dataset revealed that although extreme increase (z-score > 3.0) in enzyme expressions were observed for 42 cases, extreme decrease (z-score < -3.0) were observed for 19 cases in the 14 strains. Among them, the compensation by an extreme increase of an isozyme was observed for 4 cases, namely, Hxk1 in hxk2Δ, Idh2 in idh1Δ (two subunits of mitochondrial NAD(+)-dependent isocitrate dehydrogenase), Tkl2 in tkl1Δ (two isoforms of transketolase), and Pdc5 in pdc1Δ strains. The auto-regulation of isozymes was reported for the suppression of *PDC5* expression by Pdc1 [22]. Other cases were found to be the modulation of distant enzymes, such as over-expression of Fba1 (Fructose 1,6-bisphosphate aldolase) in the pda1Δ strain, which was lacking the E1 α subunit of the pyruvate dehydrogenase (PDH) complex (Fig 7c). Distant modulations of larger numbers of enzymes were frequently observed for the mutant strains with reduced cell growth phenotypes such as gnd1Δ, rpe1Δ, tkl1Δ, zwf1Δ (lacking the pentose phosphate pathway related genes), and pda1Δ. For example, gnd1Δ showed a slow growth phenotype (Fig 2e). This was because the loss of Gnd1 is too critical to be backed up by modulation of other enzyme abundance profiles, i.e., an extreme increase and/or decrease of distant enzyme levels. The metabolome analysis of gnd1Δ also showed that the loss of Gnd1 caused an accumulation of its substrate, 6-phosphogluconate (6PG), and a drastic perturbation in other metabolite levels (Fig 4). These results suggest that extreme regulations of enzyme expressions work in a mutant-specific manner, although detailed roles and mechanisms in the control of central carbon metabolism are unknown.

## Conclusion

The targeted proteome analysis successfully produced an enzyme expression dataset from the 30 *S*. *cerevisiae* wild type and mutant strains. The data analysis uncovered that functional compensation of the deficient enzyme was attained by using more resources for protein biosynthesis (Fig 5a). However, it was also suggested that available resources for enzyme biosynthesis in central carbon metabolism were not abundant in *S*. *cerevisiae* cells. The enzyme-enzyme co-abundance network showed that at least 30% of the variations in the enzyme abundances were explained by coordinated and global regulation, one of the most frequently observed responses being the increase in trehalose metabolism related enzymes (Figs 6 and 7a). The remaining variations should be derived from mutant-specific and local mechanisms (Fig 9).

The comparison of the enzyme abundance profile also suggest that the mutant strains could be classified into four groups. First group includes gpd1Δ strains (Fig 2c), whose enzyme abundance profiles and specific growth rates were not changed by deletions of *GPD1* genes (Fig 3). The second group forms a cluster in the center of PCA plot (Fig 6a), whose specific growth rates were similar to that of wild type (cit1Δ, eno1Δ, fbp1Δ, gpm2Δ, hor2Δ, hxk1Δ, idh1Δ, idh2Δ, idp1Δ, mae1Δ, mdh1Δ, mdh2Δ, pdc1Δ, pyc2Δ, tal1Δ, tdh1Δ, and tdh2Δ) (Fig 3). In the strains, the increase in trehalose metabolism related enzymes were commonly observed (S1 Fig). These suggested that metabolic homeostasis was maintained via the accumulation of trehalose and the regulation of glycolysis stability by the role of the trehalose shunt [44, 45]. Specific regulation of a small number of enzymes was also observed, such as the extreme increase in Pdc5 abundance in pdc1Δ (Fig 9).

A third group consists of hxk2Δ (Fig 2b), lpd1Δ, pfk1Δ (Fig 2f), and tps2Δ, in which the functions of deleted enzymes were recovered by additional modulation of enzyme abundance such as the Gcr1/2 dependent regulation of glycolytic enzymes. These strains also activated a strain-specific regulation for the extreme regulation of several enzymes (Fig 9). As demonstrated for pfk1Δ, these modulation of the enzyme abundance profile successfully compensated the functions of deleted gene and maintained metabolic homeostasis to keep cell growth (Figs 3 and 4).

A fourth group showed slower specific growth rate including gnd1Δ (Fig 2e), pda1Δ, rpe1Δ, tdh3Δ (Fig 2d), tkl1Δ, and zwf1Δ. In these mutants, the single gene deletions were too critical to be backed up by modulation of other enzyme expression profile, with an extreme increase and decrease of distant enzyme levels (Fig 9). For the case of gnd1Δ and rpe1Δ strains, an additional resource was allocated for protein biosynthesis to activate the metabolic function. Despite the employment of all possible means, however, the metabolic profile analysis showed that the metabolic homeostasis was hardly kept in the gnd1Δ strain (Fig 4). These results showed that global and local regulation of enzyme abundance levels shape central carbon metabolism in *S*. *cerevisiae* by using a limited resource for protein biosynthesis.

Although targeted proteomics is an essential tool for further dissection of the complex behavior of central carbon metabolism, the molecular mechanisms underlying the regulation of enzyme abundance, as well as their effects on metabolite accumulation and flux, need further investigation. For example, it was expected that a lack of either Zwf1 (glucose-6-phosphate dehydrogenase) or Gnd1 (a major isoform of 6-phosphogluconate dehydrogenase) would have a similar impact on metabolism, because these enzymes are both responsible for the oxidative pentose phosphate pathway (Fig 1). However, the score plot of PCA showed that the zwf1Δ and gnd1Δ strains each had different enzyme abundance profiles (Fig 6a). This could be explained by stating that the accumulation of 6-phosphogluconate in gnd1Δ (Fig 4) perturbed *S*. *cerevisiae* metabolism, for instance, via an allosteric inhibition of glucose-6-phosphate isomerase [53]. Furthermore, metabolite analysis showed that the metabolite levels, for instance, S7P accumulation in the pfk1Δ strain, were not simply explained by the enzyme abundance (Fig 4). These results show that an interaction between enzyme abundance and other layers of metabolism, such as metabolic flux, metabolite accumulation, and mRNA expression, has to be investigated considering the various post-translational regulation mechanisms of enzymes [5, 6, 54, 55].

## Materials and methods

### Yeast strains and growth conditions

*S*. *cerevisiae* strains, including S288C (MATα SUC2), BY4742 (MATα, his3Δ1, leu2Δ0, lys2Δ0, ura3Δ0), and the single gene deletion mutants were purchased from Thermo Scientific (Pittsburgh, PA, USA) (S1 Table). All strains were cultured in yeast extract peptone dextrose (YPD) medium (1% bacto yeast extract, 2% bacto peptone, 2% glucose) and synthetic dextrose (SD) medium (6.7% yeast nitrogen base without amino acids and 5% non-labeled or [U-^13^C] glucose) containing required amino acids. [U-^13^C] glucose (99%) was purchased from Cambridge Isotope Laboratories (Andover, MA, USA).

*S*. *cerevisiae* cells from glycerol stocks were cultured in 5 mL of YPD medium at 30°C for 24 h with shaking at 120 rpm. The cells were inoculated into 100 mL of SD medium in 500-mL Sakaguchi flasks and cultured at 30°C for 24 h with shaking at 120 rpm. The precultures were transferred to the main culture (100 mL of SD medium in 500-mL Sakaguchi flasks at 30°C for 48 h with shaking at 120 rpm). The initial OD_660_ values for pre- and main cultures were set at 0.2. For the preparation of isotope-labeled standards from the S288C strain, SD medium containing [U-^13^C] glucose was used for the pre- and main cultures. The BY4742 and single gene deletion strains were cultured in SD medium containing unlabeled glucose. For 96 wells cultivation, the cells were inoculated into 5 mL of SD medium and cultured at 30°C with shaking at 120 rpm. The precultures were transferred to 200 μL of SD medium in 96-well plate (Costar 3595, Corning, NY, USA) containing 0.1 mg/mL of TTA. The initial OD_600_ was set at 0.1. The plates were incubated using the plate reader (Synergy HTX, Biotek instruments, VT, USA) at 30°C for 48 h with shake mode: orbital frequency: 237cpm (4mm), and orbital speed: slow.

### Targeted proteome analysis using ultra fast mass spectrometry

Crude proteins were extracted from *S*. *cerevisiae* cells in the exponential growth phase (OD_600_ at 1.0), as previously described [9, 27], and protein concentrations were determined by the Bradford method. Trypsin digestion was performed as described previously [56, 57], and the resulting peptide solutions were desalted using GL-Tip GC micropipette tips (GL Science, Tokyo, Japan). In this manner, 60 μL of digested peptides were prepared from samples containing 50 μg of crude protein. Equal amounts of digested peptide derived from the target (cultured using nonlabeled glucose) and S288C (cultured in [U-^13^C] glucose medium as an internal standard) strains were mixed [13]. An identical ^13^C labeled peptide mixtures prepared from S288C strain was employed for all analyses in this study. The peptide samples (2 μL) were analyzed using a nano-LC-UFMS system (LC-20ADnano and LCMS-8040, Shimadzu, Kyoto, Japan), equipped with a valve system (FCV nano, Shimadzu), a nanospray interface (N8040, AMR, Tokyo, Japan), and a spray tip (Fortis tip 150–20, AMR). The analytical conditions were as follows. HPLC: column: L-column ODS (pore size: 5 μm, 0.1 × 150 mm, CERI, Tokyo Japan); trap column: L-column ODS (pore size: 5 μm, 0.3 × 5 mm, CERI); solvent system: 0.1% formic acid and 5% acetonitrile in water: 0.1% formic acid and 95% acetonitrile in water; gradient program: 0:100, v/v at 0 min; 0:100 at 7 min; 35:65 at 45 min; 50:50 at 50 min; and 100:0 at 65 min; flow rate: 400 nL/min. MS detection: interface temperature: 350°C; DL temperature: 150°C; heat block temperature: 200°C; drying gas flow: off; CID gas pressure: 310; interface voltage: +1.6 keV; detection mode: MRM positive. The data were recorded with the aid of LabSolutions LCMS version 5.6 (Shimadzu). The MRM assay method for the enzyme analysis of yeast central metabolism, described by Picotti et al. [9, 27], was used with modifications (S2 Table).

### Data analysis

Chromatographic data was processed using Skyline version 3.1 [58, 59]. Relative peak areas were determined for all peptides using the ^13^C labeled peptides as the internal standards. In order to correct a systematic error derived from the protein quantification by the Bradford method, 55 proteins with smaller expression variations among 87 samples were selected considering relative standard deviations. Signal intensities of enzymes were normalized by dividing a mean relative peak area of 55 proteins of the samples. All data analysis was performed using the Multi experiment viewer (MeV) version 4.8 [60] and in-house scripts written in Perl. Statistical tests (Student’s t test) were performed for the raw abundances data. For principle component analysis, the mean abundances were converted to log 2 transformed and normalized data by using the functions of MeV. For the mutant-specific regulation of enzyme expression levels, the abundances were z-scored by subtracting the mean of 30 strains and then dividing by the standard deviation. The data obtained from gcr2Δ and mutant strains of the target protein (for example, Pdc1 expression data in pdc1Δ strains) were not used for the calculation of spearman correlation coefficients and relative standard deviations. The enzyme expression profiles and co-expression networks were visualized by Cytoscape3.0 [61, 62] and VANTED v2.2.0 [63].

### LC-MS/MS analysis of intermediate metabolites

Culture broth was sampled rapidly and filtered through a 0.5-μm pore size filter (PTFE-type membrane, ADVANTEC, Japan). Cells on the filter were immediately immersed in 1.6 mL methanol (-80°C) and kept at -80°C until extraction. Following addition of 1.6 mL of chloroform (-30°C) and 640 μL of Milli-Q water (4°C) and vortexing for 1 min, the mixture was centrifuged at 15,000 g for 5 min at 4°C. Two hundred and fifty microliters of the aqueous layer was transferred to a 1.5-mL tube and dried using a SpeedVac SPD1010 (Thermo Scientific, Japan) at room temperature. The dried samples were suspended in 50 μL of Milli-Q water. LC-MS/MS analysis (LC: Agilent 1100 series; Agilent Technologies, MS/MS: API 2000; MA, AB SCIEX) was performed using the previously described method (17). The peak of each target metabolite was identified by comparing its chromatographic behavior with that of an authentic standard. Peak area was determined using the software Analyst (version 1.6.2, AB SCIEX).

## Supporting information

S1 Fig Protein abundance profiles of the central metabolism related enzymes in *Saccharomyces cerevisiae*. (PDF)

S2 Fig Protein investment for the biosynthesis of 110 enzymes. (PDF)

S3 Fig The composition of enzyme proteins. (PDF)

S4 Fig Distributions of Spearman's rank-order correlation coefficients across all enzyme. (PDF)

S1 Table List of single gene deletion mutants, those specific growth rates and numbers of significantly increased and decreased enzymes. (XLSX)

S2 Table SRM assay methods. (XLSX)

S3 Table Relative enzyme expression levels. (XLSX)
