# Supplementary material for: Targeted proteome analysis of single-gene deletion strains of Saccharomyces cerevisiae lacking enzymes in the central carbon metabolism
Source: PLoS One. 2017 Feb 27;12(2):e0172742. doi: 10.1371/journal.pone.0172742 (PMC5328394; doi:10.1371/journal.pone.0172742)
Supplement: S1 Fig — (PDF) [file pone.0172742.s001.pdf]

S1 Fig

Protein abundance profiles of the central metabolism related enzymes in *Saccharomyces cerevisiae*

Enzyme abundance levels relative to that of wild type (BY4742) were shown as a heat map of simplified metabolite pathways.

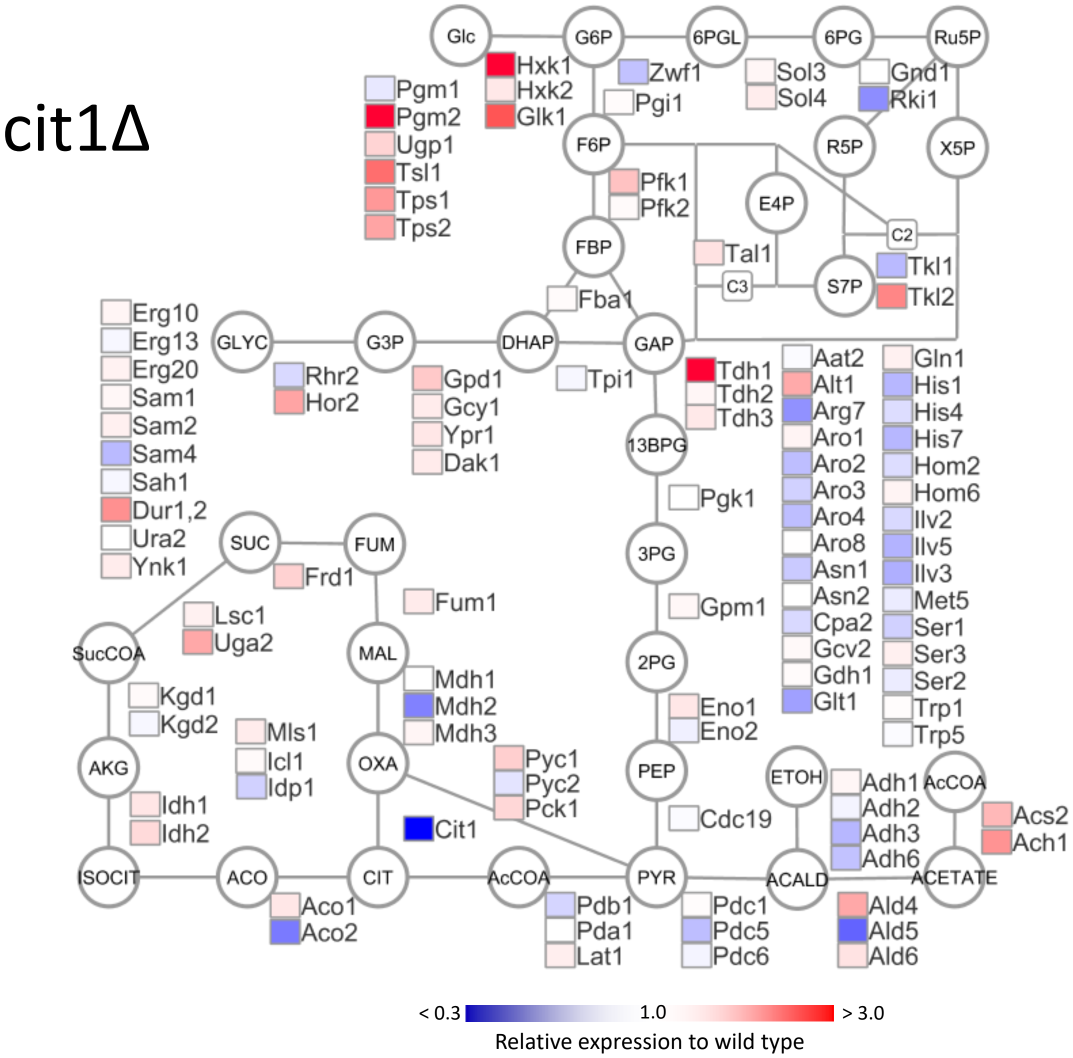

eno1Δ

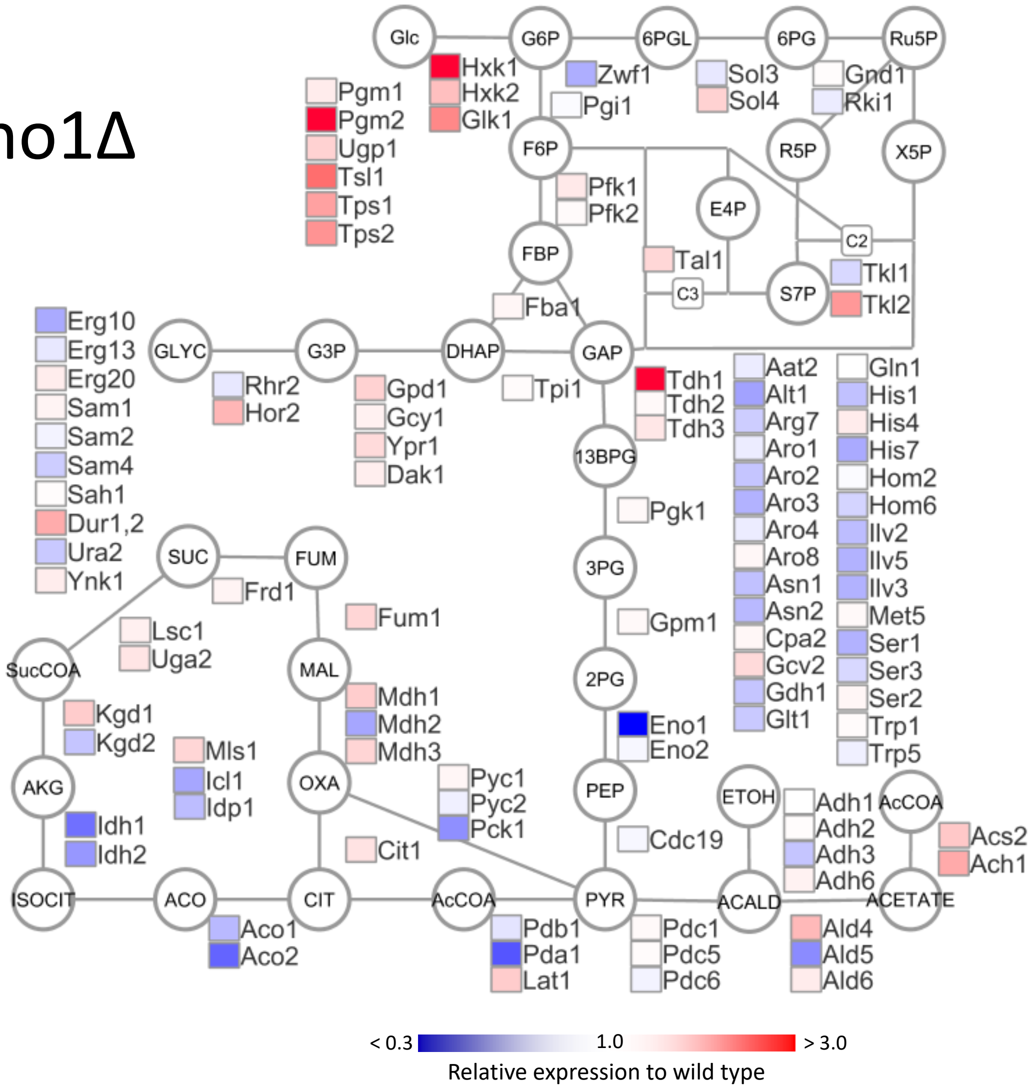
Glc
G6P
6PGL
6PG
Ru5P
Hxk1
Hxk2
Glk1
Pgm1
Pgm2
Ugp1
Tsl1
Tps1
Tps2
Zwf1
Pgi1
Sol3
Sol4
Gnd1
Rki1
F6P
R5P
X5P
Pfk1
Pfk2
E4P
FBP
C2
Tal1
Tkl1
Tkl2
C3
S7P
Fba1
Erg10
Erg13
Erg20
Sam1
Sam2
Sam4
Sah1
Dur1,2
Ura2
Ynk1
GLYC
G3P
DHAP
GAP
Rhr2
Hor2
Gpd1
Gcy1
Ypr1
Dak1
Tpi1
Tdh1
Tdh2
Tdh3
13BPG
Pgk1
3PG
Gpm1
2PG
Eno1
Eno2
PEP
Cdc19
PYR
Aat2
Alt1
Arg7
Aro1
Aro2
Aro3
Aro4
Aro8
Asn1
Asn2
Cpa2
Gcv2
Gdh1
Glt1
Gln1
His1
His4
His7
Hom2
Hom6
Ilv2
Ilv5
Ilv3
Met5
Ser1
Ser3
Ser2
Trp1
Trp5
SUC
FUM
Frd1
Fum1
SucCOA
Lsc1
Uga2
MAL
Mdh1
Mdh2
Mdh3
Kgd1
Kgd2
Mls1
Icl1
Idp1
AKG
OXA
Pyc1
Pyc2
Pck1
Idh1
Idh2
Cit1
ISOCIT
ACO
CIT
AcCOA
Aco1
Aco2
Pdb1
Pda1
Lat1
ETOH
Adh1
Adh2
Adh3
Adh6
AcCOA
Acs2
Ach1
ACALD
ACETATE
Pdc1
Pdc5
Pdc6
Ald4
Ald5
Ald6
< 0.3
1.0
> 3.0
Relative expression to wild type

fbp1Δ

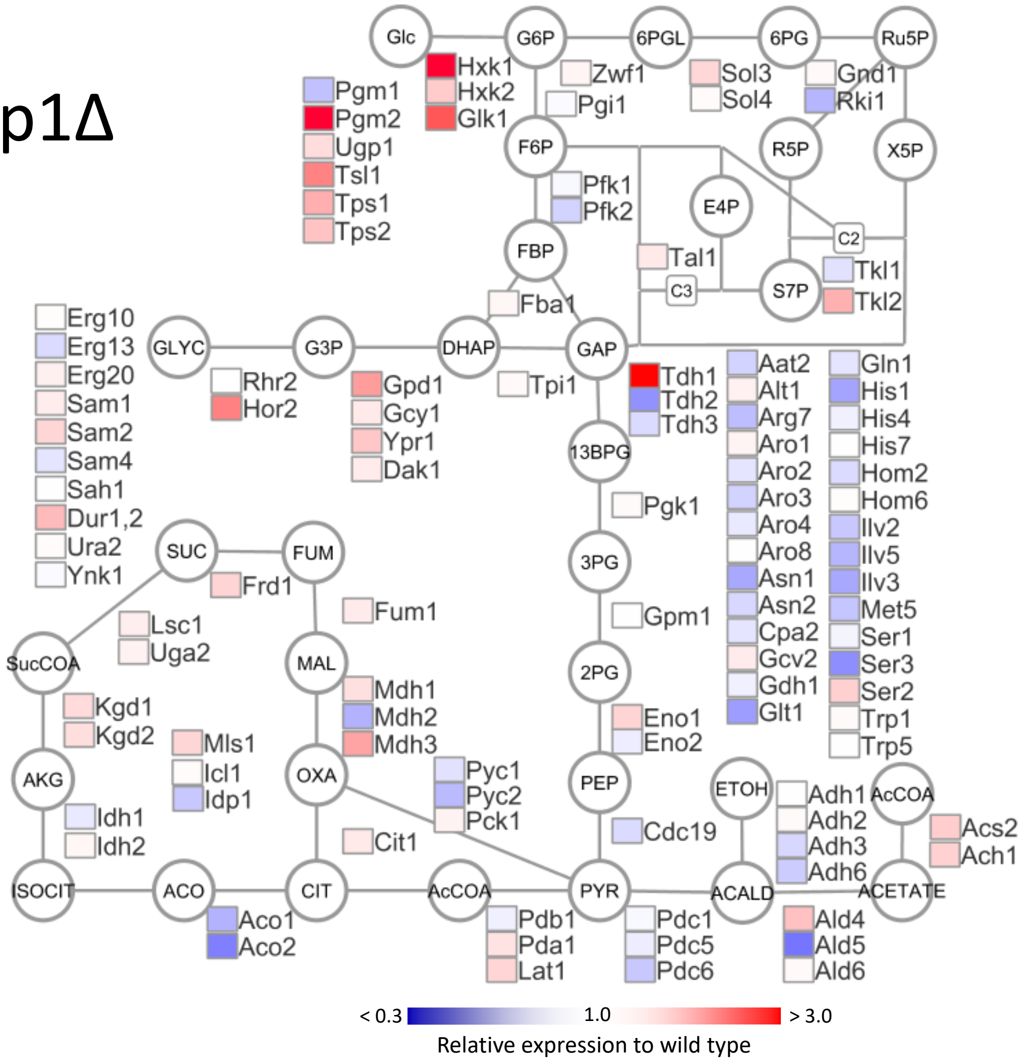

Glc
G6P
6PGL
6PG
Ru5P
Hxk1
Hxk2
Glk1
Zwf1
Pgi1
Sol3
Sol4
Gnd1
Rki1
Pgm1
Pgm2
Ugp1
Tsl1
Tps1
Tps2
F6P
R5P
X5P
Pfk1
Pfk2
E4P
C2
FBP
Tal1
C3
S7P
Tkl1
Tkl2
Fba1
Erg10
Erg13
Erg20
Sam1
Sam2
Sam4
Sah1
Dur1,2
Ura2
Ynk1
GLYC
G3P
DHAP
GAP
Rhr2
Hor2
Gpd1
Gcy1
Ypr1
Dak1
Tpi1
Tdh1
Tdh2
Tdh3
13BPG
Pgk1
3PG
Gpm1
2PG
Eno1
Eno2
PEP
Cdc19
Aat2
Alt1
Arg7
Aro1
Aro2
Aro3
Aro4
Aro8
Asn1
Asn2
Cpa2
Gcv2
Gdh1
Glt1
Gln1
His1
His4
His7
Hom2
Hom6
Ilv2
Ilv5
Ilv3
Met5
Ser1
Ser3
Ser2
Trp1
Trp5
SUC
FUM
Frd1
Fum1
SucCOA
Lsc1
Uga2
MAL
Kgd1
Kgd2
Mdh1
Mdh2
Mdh3
Mls1
Icl1
Idp1
AKG
OXA
Pyc1
Pyc2
Pck1
Idh1
Idh2
Cit1
ETOH
Adh1
Adh2
Adh3
Adh6
AcCOA
Acs2
Ach1
ISOCIT
ACO
CIT
AcCOA
PYR
ACALD
ACETATE
Aco1
Aco2
Pdb1
Pda1
Lat1
Pdc1
Pdc5
Pdc6
Ald4
Ald5
Ald6
< 0.3
1.0
> 3.0
Relative expression to wild type

gcr2Δ

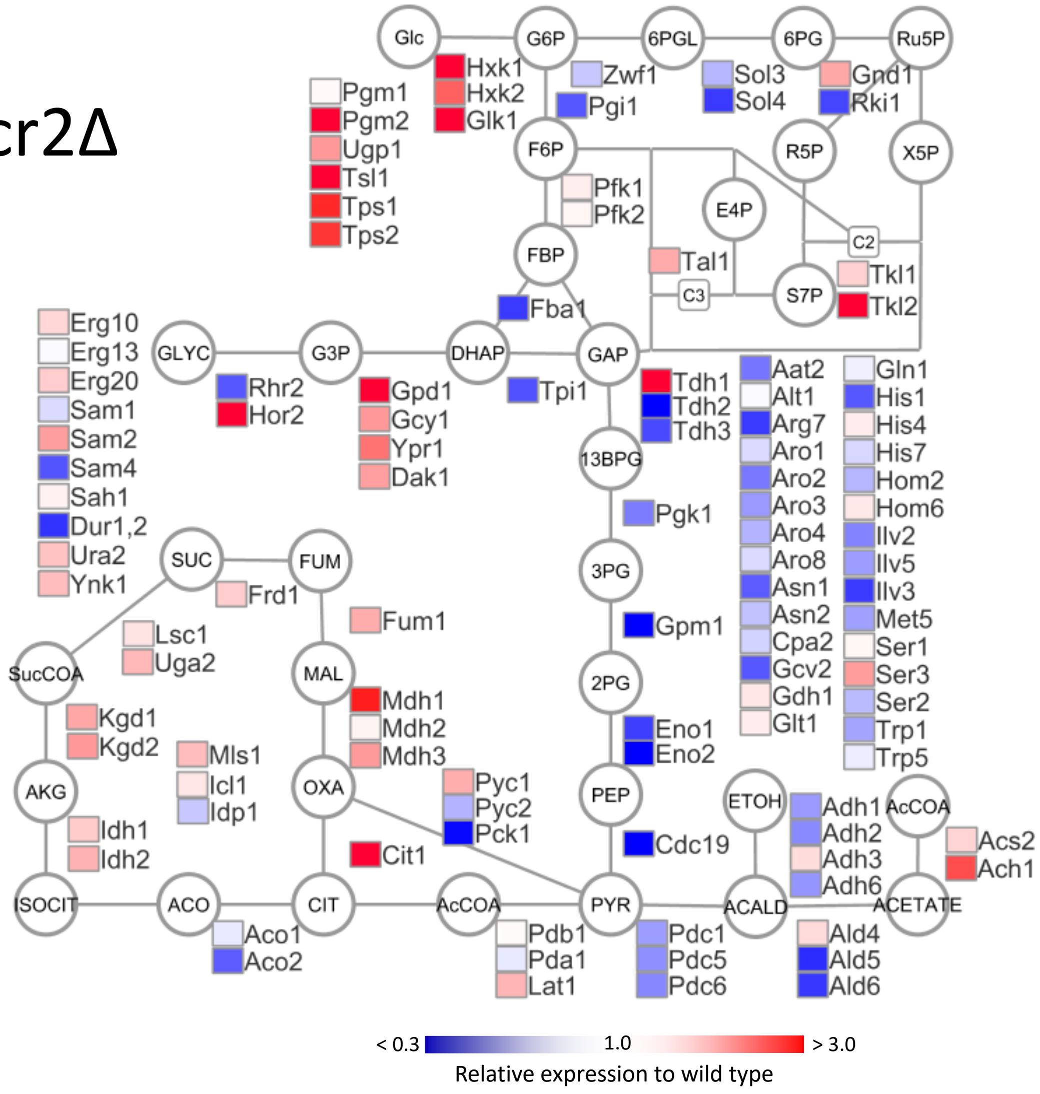
Glc
G6P
6PGL
6PG
Ru5P
Hxk1
Hxk2
Glk1
Pgm1
Pgm2
Ugp1
Tsl1
Tps1
Tps2
Zwf1
Pgi1
Sol3
Sol4
Gnd1
Rki1
F6P
R5P
X5P
Pfk1
Pfk2
E4P
C2
FBP
Tal1
C3
S7P
Tkl1
Tkl2
Fba1
Erg10
Erg13
Erg20
Sam1
Sam2
Sam4
Sah1
Dur1,2
Ura2
Ynk1
GLYC
G3P
DHAP
GAP
Rhr2
Hor2
Gpd1
Gcy1
Ypr1
Dak1
Tpi1
Tdh1
Tdh2
Tdh3
13BPG
Aat2
Alt1
Arg7
Aro1
Aro2
Aro3
Aro4
Aro8
Asn1
Asn2
Cpa2
Gcv2
Gdh1
Glt1
Gln1
His1
His4
His7
Hom2
Hom6
Ilv2
Ilv5
Ilv3
Met5
Ser1
Ser3
Ser2
Trp1
Trp5
Pgk1
3PG
Gpm1
2PG
Eno1
Eno2
PEP
Cdc19
SUC
FUM
Frd1
Fum1
Lsc1
Uga2
SucCOA
MAL
Mdh1
Mdh2
Mdh3
Kgd1
Kgd2
Mls1
Icl1
Idp1
AKG
OXA
Pyc1
Pyc2
Pck1
Idh1
Idh2
Cit1
ETOH
Adh1
Adh2
Adh3
Adh6
AcCOA
Acs2
Ach1
ISOCIT
ACO
CIT
AcCOA
PYR
ACALD
ACETATE
Aco1
Aco2
Pdb1
Pda1
Lat1
Pdc1
Pdc5
Pdc6
Ald4
Ald5
Ald6
< 0.3
1.0
> 3.0
Relative expression to wild type

gnd1Δ

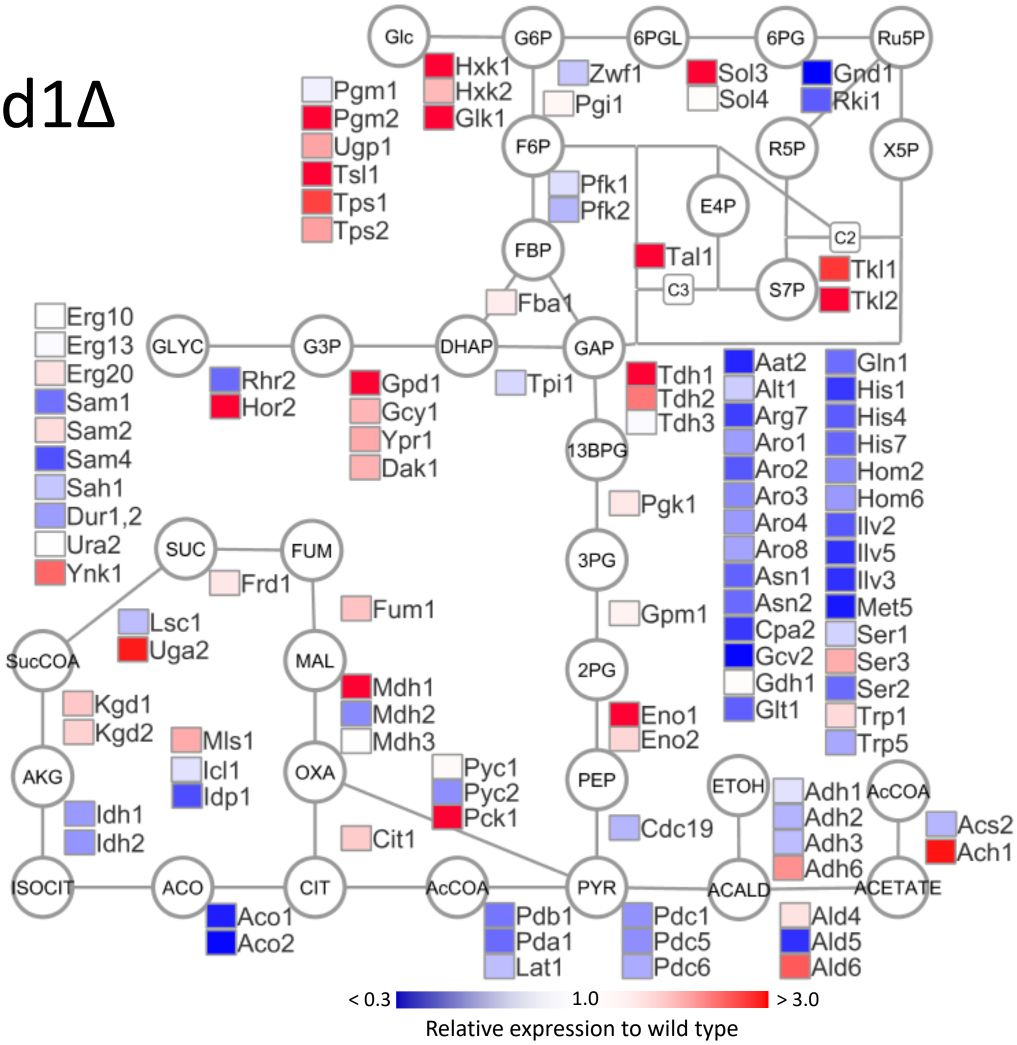
Glc
G6P
6PGL
6PG
Ru5P
Hxk1
Hxk2
Glk1
Zwf1
Pgi1
Sol3
Sol4
Gnd1
Rki1
Pgm1
Pgm2
Ugp1
Tsl1
Tps1
Tps2
F6P
R5P
X5P
Pfk1
Pfk2
E4P
C2
FBP
Tal1
C3
S7P
Tkl1
Tkl2
Fba1
Erg10
Erg13
Erg20
Sam1
Sam2
Sam4
Sah1
Dur1,2
Ura2
Ynk1
GLYC
G3P
DHAP
GAP
Rhr2
Hor2
Gpd1
Gcy1
Ypr1
Dak1
Tpi1
Tdh1
Tdh2
Tdh3
13BPG
Pgk1
3PG
Gpm1
2PG
Eno1
Eno2
PEP
Cdc19
Aat2
Alt1
Arg7
Aro1
Aro2
Aro3
Aro4
Aro8
Asn1
Asn2
Cpa2
Gcv2
Gdh1
Glt1
Gln1
His1
His4
His7
Hom2
Hom6
Ilv2
Ilv5
Ilv3
Met5
Ser1
Ser3
Ser2
Trp1
Trp5
SUC
FUM
Frd1
Fum1
Lsc1
Uga2
SucCOA
MAL
Mdh1
Mdh2
Mdh3
Kgd1
Kgd2
Mls1
Icl1
Idp1
AKG
OXA
Pyc1
Pyc2
Pck1
Idh1
Idh2
Cit1
ETOH
Adh1
Adh2
Adh3
Adh6
AcCOA
Acs2
Ach1
ISOCIT
ACO
CIT
AcCOA
PYR
ACALD
ACETATE
Aco1
Aco2
Pdb1
Pda1
Lat1
Pdc1
Pdc5
Pdc6
Ald4
Ald5
Ald6
< 0.3
1.0
> 3.0
Relative expression to wild type

gpd1Δ

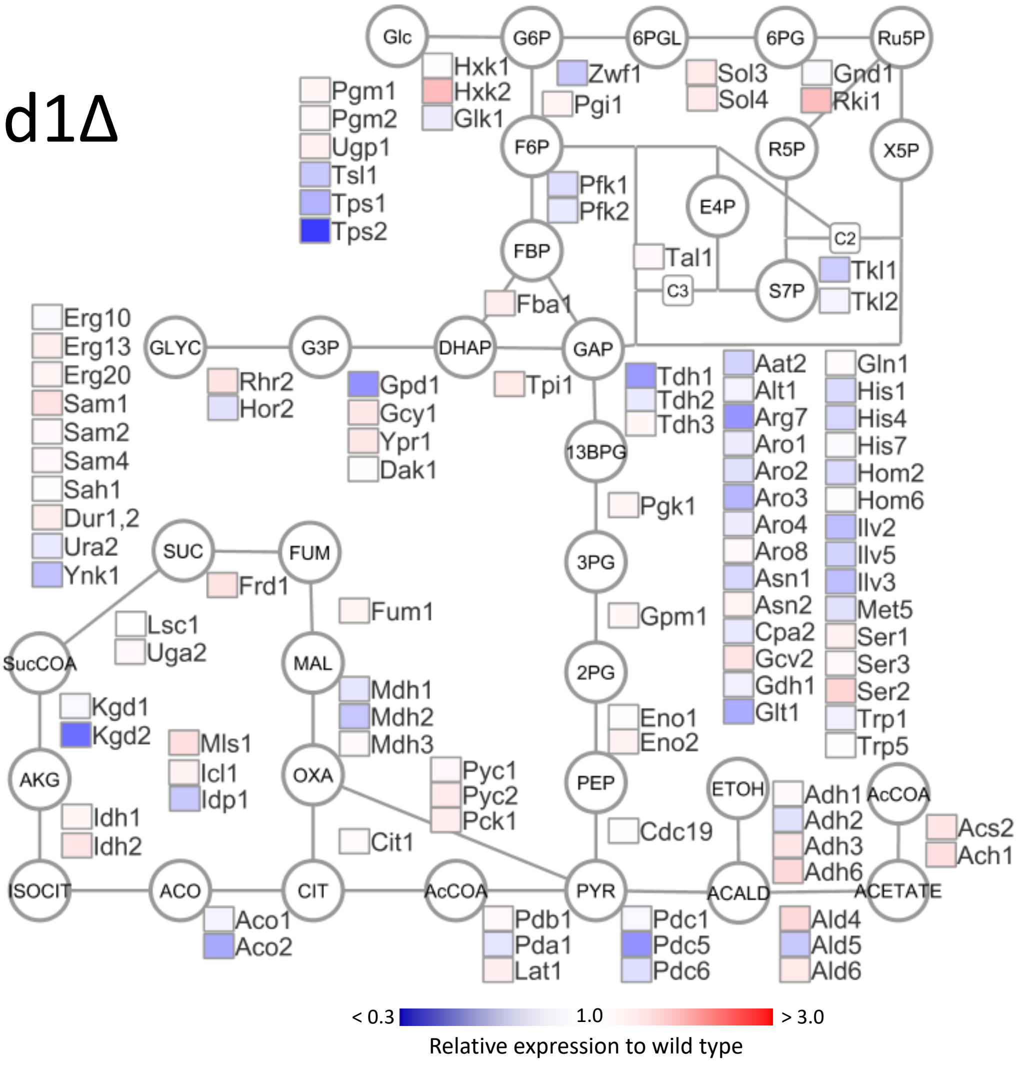
Glc
G6P
6PGL
6PG
Ru5P
Hxk1
Hxk2
Glk1
Zwf1
Pgi1
Sol3
Sol4
Gnd1
Rki1
Pgm1
Pgm2
Ugp1
Tsl1
Tps1
Tps2
F6P
R5P
X5P
Pfk1
Pfk2
E4P
C2
FBP
Tal1
C3
S7P
Tkl1
Tkl2
Fba1
Erg10
Erg13
Erg20
Sam1
Sam2
Sam4
Sah1
Dur1,2
Ura2
Ynk1
GLYC
G3P
DHAP
GAP
Rhr2
Hor2
Gpd1
Gcy1
Ypr1
Dak1
Tpi1
Tdh1
Tdh2
Tdh3
13BPG
Aat2
Alt1
Arg7
Aro1
Aro2
Aro3
Aro4
Aro8
Asn1
Asn2
Cpa2
Gcv2
Gdh1
Glt1
Gln1
His1
His4
His7
Hom2
Hom6
Ilv2
Ilv5
Ilv3
Met5
Ser1
Ser3
Ser2
Trp1
Trp5
Pgk1
3PG
Gpm1
2PG
Eno1
Eno2
PEP
Cdc19
SUC
FUM
Frd1
Fum1
Lsc1
Uga2
SucCOA
MAL
Mdh1
Mdh2
Mdh3
Kgd1
Kgd2
AKG
Mls1
Icl1
Idp1
OXA
Pyc1
Pyc2
Pck1
Idh1
Idh2
Cit1
ETOH
Adh1
AcCOA
Adh2
Adh3
Adh6
Acs2
Ach1
ISOCIT
ACO
CIT
AcCOA
PYR
ACALD
ACETATE
Aco1
Aco2
Pdb1
Pda1
Lat1
Pdc1
Pdc5
Pdc6
Ald4
Ald5
Ald6
< 0.3
1.0
> 3.0
Relative expression to wild type

gpm2Δ

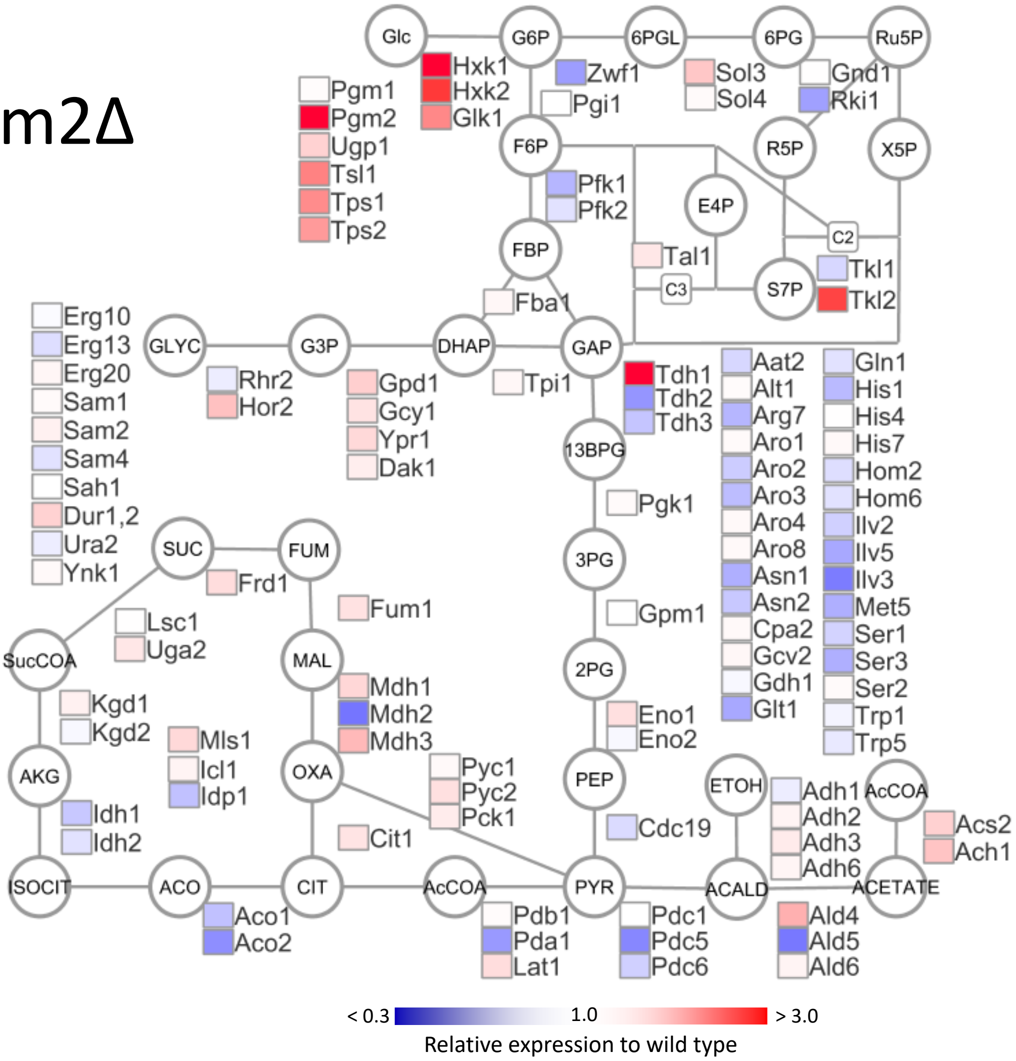
Glc
G6P
6PGL
6PG
Ru5P
Hxk1
Hxk2
Glk1
Zwf1
Pgi1
Sol3
Sol4
Gnd1
Rki1
Pgm1
Pgm2
Ugp1
Tsl1
Tps1
Tps2
F6P
R5P
X5P
Pfk1
Pfk2
E4P
C2
FBP
Tal1
C3
S7P
Tkl1
Tkl2
Fba1
Erg10
Erg13
Erg20
Sam1
Sam2
Sam4
Sah1
Dur1,2
Ura2
Ynk1
GLYC
G3P
DHAP
GAP
Rhr2
Hor2
Gpd1
Gcy1
Ypr1
Dak1
Tpi1
Tdh1
Tdh2
Tdh3
13BPG
Pgk1
3PG
Gpm1
2PG
Eno1
Eno2
PEP
Cdc19
Aat2
Alt1
Arg7
Aro1
Aro2
Aro3
Aro4
Aro8
Asn1
Asn2
Cpa2
Gcv2
Gdh1
Glt1
Gln1
His1
His4
His7
Hom2
Hom6
Ilv2
Ilv5
Ilv3
Met5
Ser1
Ser3
Ser2
Trp1
Trp5
SUC
FUM
Frd1
Fum1
Lsc1
Uga2
SucCOA
MAL
Mdh1
Mdh2
Mdh3
Kgd1
Kgd2
AKG
Mls1
Icl1
Idp1
OXA
Pyc1
Pyc2
Pck1
Idh1
Idh2
Cit1
ISOCIT
ACO
CIT
AcCOA
PYR
ETOH
ACALD
AcCOA
ACETATE
Adh1
Adh2
Adh3
Adh6
Acs2
Ach1
Aco1
Aco2
Pdb1
Pda1
Lat1
Pdc1
Pdc5
Pdc6
Ald4
Ald5
Ald6
< 0.3
1.0
> 3.0
Relative expression to wild type

hor2Δ

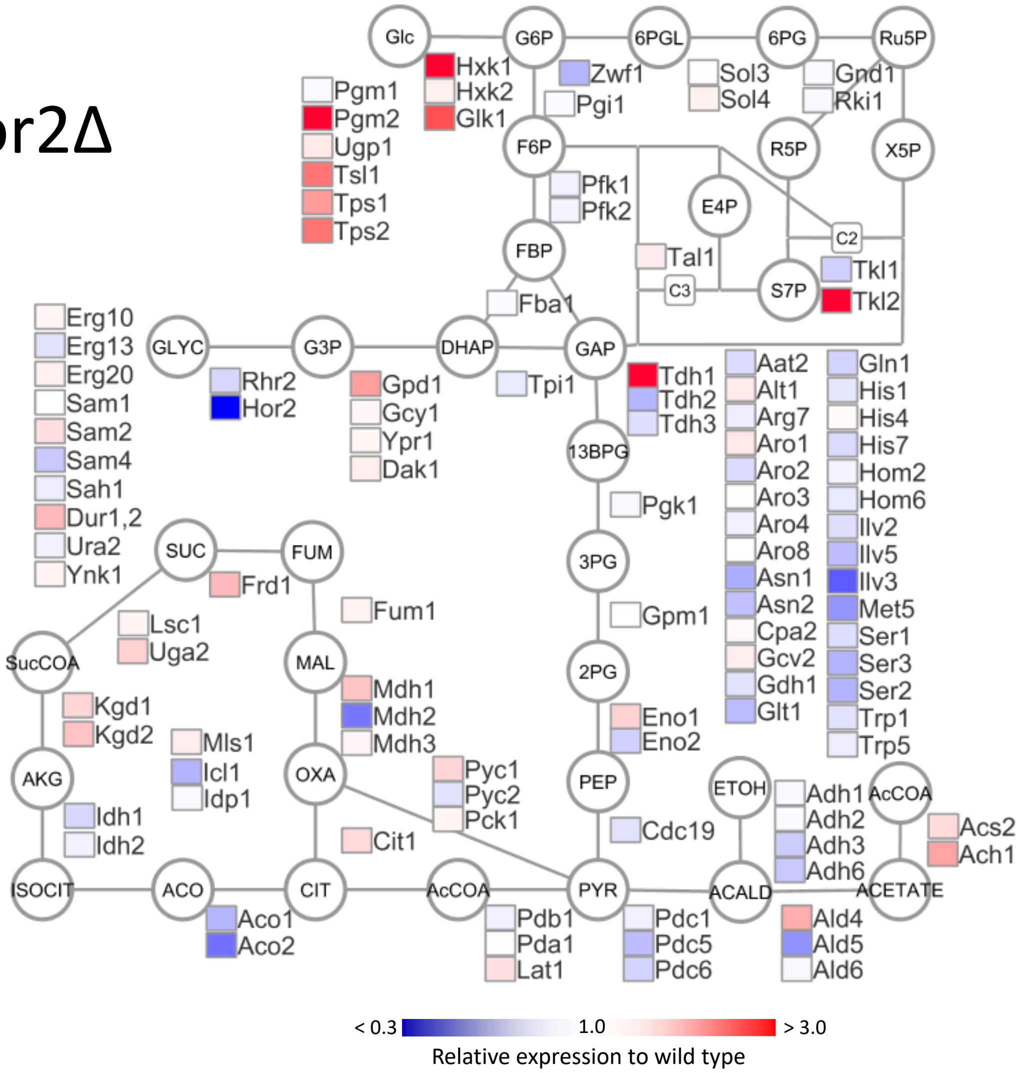

Glc
G6P
6PGL
6PG
Ru5P
Hxk1
Hxk2
Glk1
Pgm1
Pgm2
Ugp1
Tsl1
Tps1
Tps2
Zwf1
Pgi1
Sol3
Sol4
Gnd1
Rki1
F6P
R5P
X5P
Pfk1
Pfk2
E4P
C2
FBP
Tal1
C3
S7P
Tkl1
Tkl2
Fba1
Erg10
Erg13
Erg20
Sam1
Sam2
Sam4
Sah1
Dur1,2
Ura2
Ynk1
GLYC
G3P
DHAP
GAP
Rhr2
Hor2
Gpd1
Gcy1
Ypr1
Dak1
Tpi1
Tdh1
Tdh2
Tdh3
13BPG
Pgk1
3PG
Gpm1
2PG
Eno1
Eno2
PEP
Cdc19
Aat2
Alt1
Arg7
Aro1
Aro2
Aro3
Aro4
Aro8
Asn1
Asn2
Cpa2
Gcv2
Gdh1
Glt1
Gln1
His1
His4
His7
Hom2
Hom6
Ilv2
Ilv5
Ilv3
Met5
Ser1
Ser3
Ser2
Trp1
Trp5
SUC
FUM
Frd1
Fum1
SucCOA
Lsc1
Uga2
MAL
Mdh1
Mdh2
Mdh3
Kgd1
Kgd2
Mls1
Icl1
Idp1
AKG
OXA
Pyc1
Pyc2
Pck1
Idh1
Idh2
Cit1
ETOH
Adh1
Adh2
Adh3
Adh6
AcCOA
Acs2
Ach1
ISOCIT
ACO
CIT
AcCOA
PYR
ACALD
ACETATE
Aco1
Aco2
Pdb1
Pda1
Lat1
Pdc1
Pdc5
Pdc6
Ald4
Ald5
Ald6
< 0.3
1.0
> 3.0
Relative expression to wild type

hxk1Δ

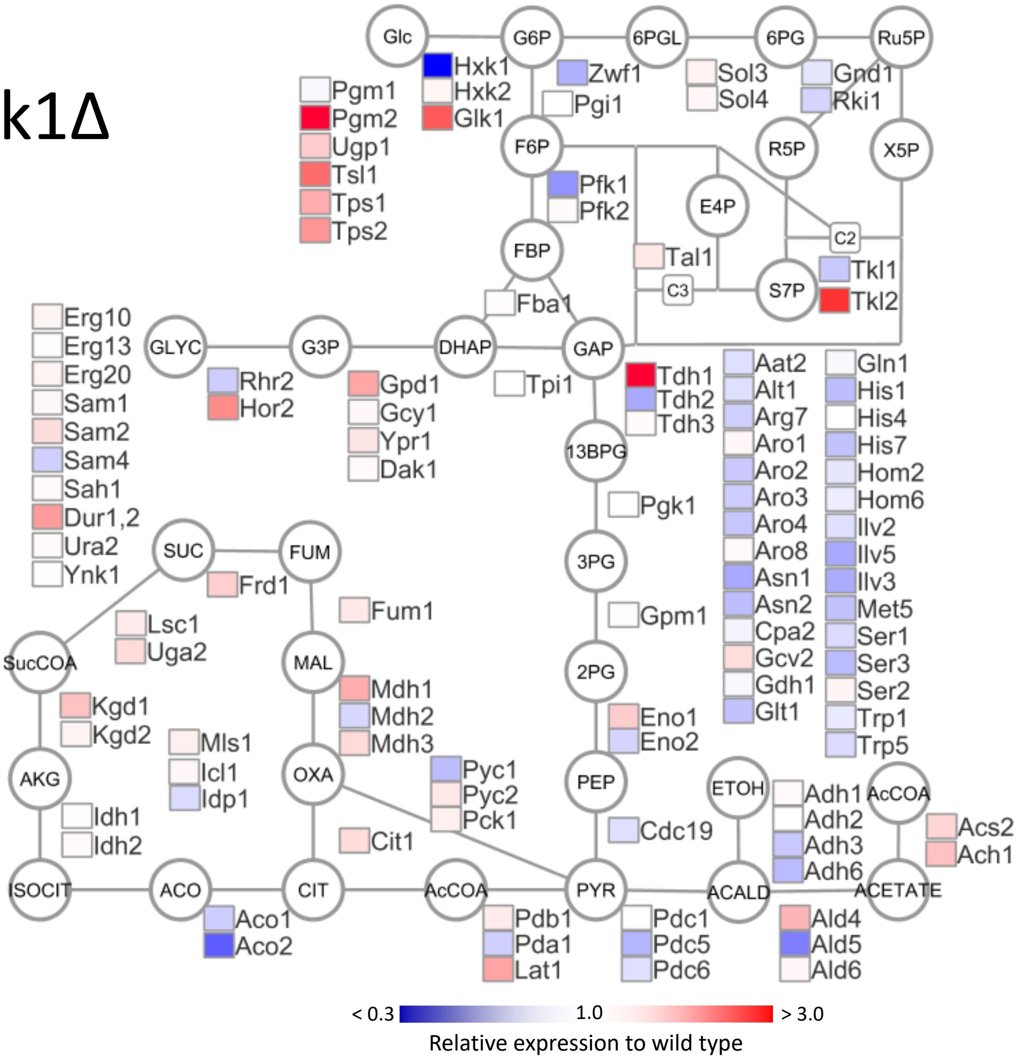
Glc
G6P
6PGL
6PG
Ru5P
Hxk1
Hxk2
Glk1
Zwf1
Pgi1
Sol3
Sol4
Gnd1
Rki1
Pgm1
Pgm2
Ugp1
Tsl1
Tps1
Tps2
F6P
R5P
X5P
Pfk1
Pfk2
E4P
C2
FBP
Tal1
C3
S7P
Tkl1
Tkl2
Fba1
Erg10
Erg13
Erg20
Sam1
Sam2
Sam4
Sah1
Dur1,2
Ura2
Ynk1
GLYC
G3P
DHAP
GAP
Rhr2
Hor2
Gpd1
Gcy1
Ypr1
Dak1
Tpi1
Tdh1
Tdh2
Tdh3
13BPG
Pgk1
3PG
Gpm1
2PG
Eno1
Eno2
PEP
Cdc19
Aat2
Alt1
Arg7
Aro1
Aro2
Aro3
Aro4
Aro8
Asn1
Asn2
Cpa2
Gcv2
Gdh1
Glt1
Gln1
His1
His4
His7
Hom2
Hom6
Ilv2
Ilv5
Ilv3
Met5
Ser1
Ser3
Ser2
Trp1
Trp5
SUC
FUM
Frd1
Fum1
SucCOA
Lsc1
Uga2
MAL
Mdh1
Mdh2
Mdh3
Kgd1
Kgd2
AKG
Mls1
Icl1
Idp1
OXA
Pyc1
Pyc2
Pck1
Idh1
Idh2
Cit1
ETOH
Adh1
Adh2
Adh3
Adh6
AcCOA
Acs2
Ach1
ISOCIT
ACO
CIT
AcCOA
PYR
ACALD
ACETATE
Aco1
Aco2
Pdb1
Pda1
Lat1
Pdc1
Pdc5
Pdc6
Ald4
Ald5
Ald6
< 0.3
1.0
> 3.0
Relative expression to wild type

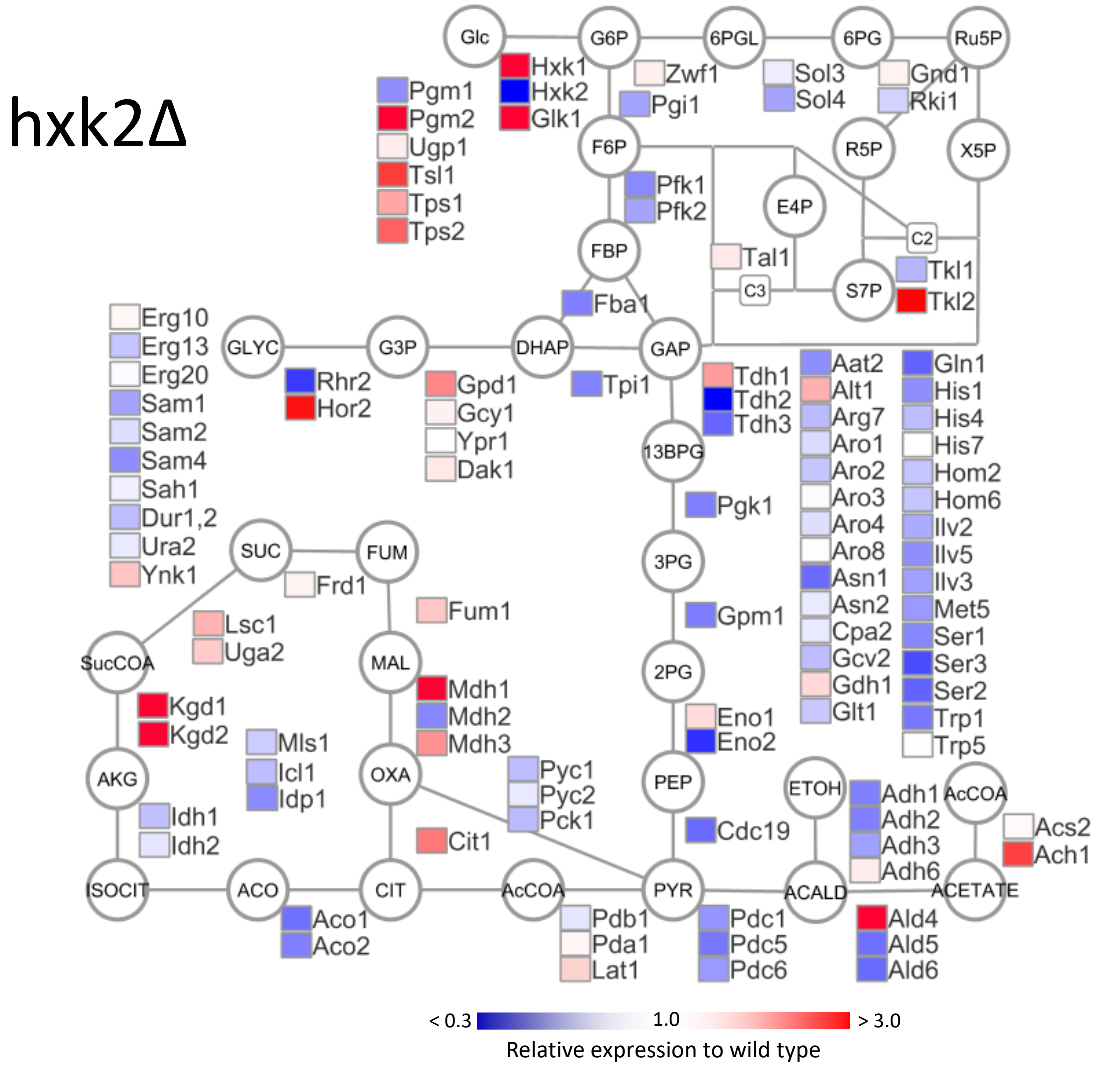
hxk2Δ
Glc
G6P
6PGL
6PG
Ru5P
Hxk1
Hxk2
Glk1
Zwf1
Sol3
Sol4
Gnd1
Rki1
Pgm1
Pgm2
Ugp1
Tsl1
Tps1
Tps2
Pgi1
F6P
R5P
X5P
Pfk1
Pfk2
E4P
C2
FBP
Tal1
C3
S7P
Tkl1
Tkl2
Fba1
Erg10
Erg13
Erg20
Sam1
Sam2
Sam4
Sah1
Dur1,2
Ura2
Ynk1
GLYC
G3P
DHAP
GAP
Rhr2
Hor2
Gpd1
Gcy1
Ypr1
Dak1
Tpi1
Tdh1
Tdh2
Tdh3
13BPG
Pgk1
3PG
Gpm1
2PG
Eno1
Eno2
PEP
Cdc19
Aat2
Alt1
Arg7
Aro1
Aro2
Aro3
Aro4
Aro8
Asn1
Asn2
Cpa2
Gcv2
Gdh1
Glt1
Gln1
His1
His4
His7
Hom2
Hom6
Ilv2
Ilv5
Ilv3
Met5
Ser1
Ser3
Ser2
Trp1
Trp5
SUC
FUM
Frd1
Fum1
Lsc1
Uga2
SucCOA
MAL
Mdh1
Mdh2
Mdh3
Kgd1
Kgd2
AKG
Mls1
Icl1
Idp1
OXA
Pyc1
Pyc2
Pck1
Idh1
Idh2
Cit1
ISOCIT
ACO
CIT
AcCOA
PYR
ETOH
ACALD
ACETATE
AcCOA
Adh1
Adh2
Adh3
Adh6
Acs2
Ach1
Aco1
Aco2
Pdb1
Pda1
Lat1
Pdc1
Pdc5
Pdc6
Ald4
Ald5
Ald6
< 0.3
1.0
> 3.0
Relative expression to wild type

idh1Δ

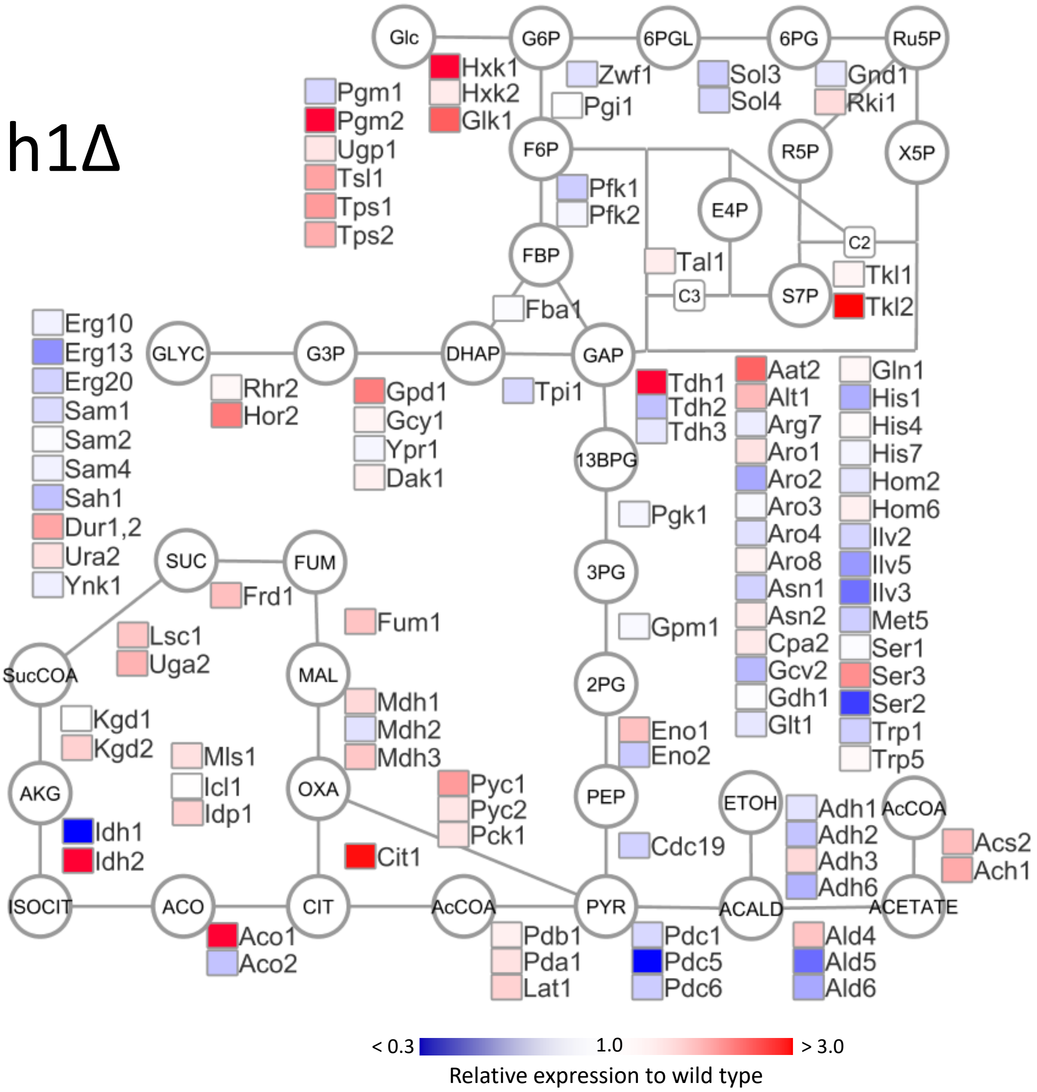

Glc
G6P
6PGL
6PG
Ru5P
Hxk1
Hxk2
Glk1
Pgm1
Pgm2
Ugp1
Tsl1
Tps1
Tps2
Zwf1
Pgi1
Sol3
Sol4
Gnd1
Rki1
F6P
R5P
X5P
Pfk1
Pfk2
E4P
C2
FBP
Tal1
Tkl1
Tkl2
S7P
C3
Fba1
Erg10
Erg13
Erg20
Sam1
Sam2
Sam4
Sah1
Dur1,2
Ura2
Ynk1
GLYC
G3P
DHAP
GAP
Rhr2
Hor2
Gpd1
Gcy1
Ypr1
Dak1
Tpi1
Tdh1
Tdh2
Tdh3
13BPG
Pgk1
3PG
Gpm1
2PG
Eno1
Eno2
PEP
Cdc19
PYR
Aat2
Alt1
Arg7
Aro1
Aro2
Aro3
Aro4
Aro8
Asn1
Asn2
Cpa2
Gcv2
Gdh1
Glt1
Gln1
His1
His4
His7
Hom2
Hom6
Ilv2
Ilv5
Ilv3
Met5
Ser1
Ser3
Ser2
Trp1
Trp5
SUC
FUM
Frd1
Fum1
Lsc1
Uga2
SucCOA
MAL
Mdh1
Mdh2
Mdh3
Kgd1
Kgd2
Mls1
Icl1
Idp1
AKG
OXA
Pyc1
Pyc2
Pck1
Idh1
Idh2
Cit1
ISOCIT
ACO
CIT
AcCOA
Aco1
Aco2
Pdb1
Pda1
Lat1
Pdc1
Pdc5
Pdc6
ETOH
Adh1
Adh2
Adh3
Adh6
AcCOA
Acs2
Ach1
ACALD
ACETATE
Ald4
Ald5
Ald6
< 0.3
1.0
> 3.0
Relative expression to wild type

idh2Δ

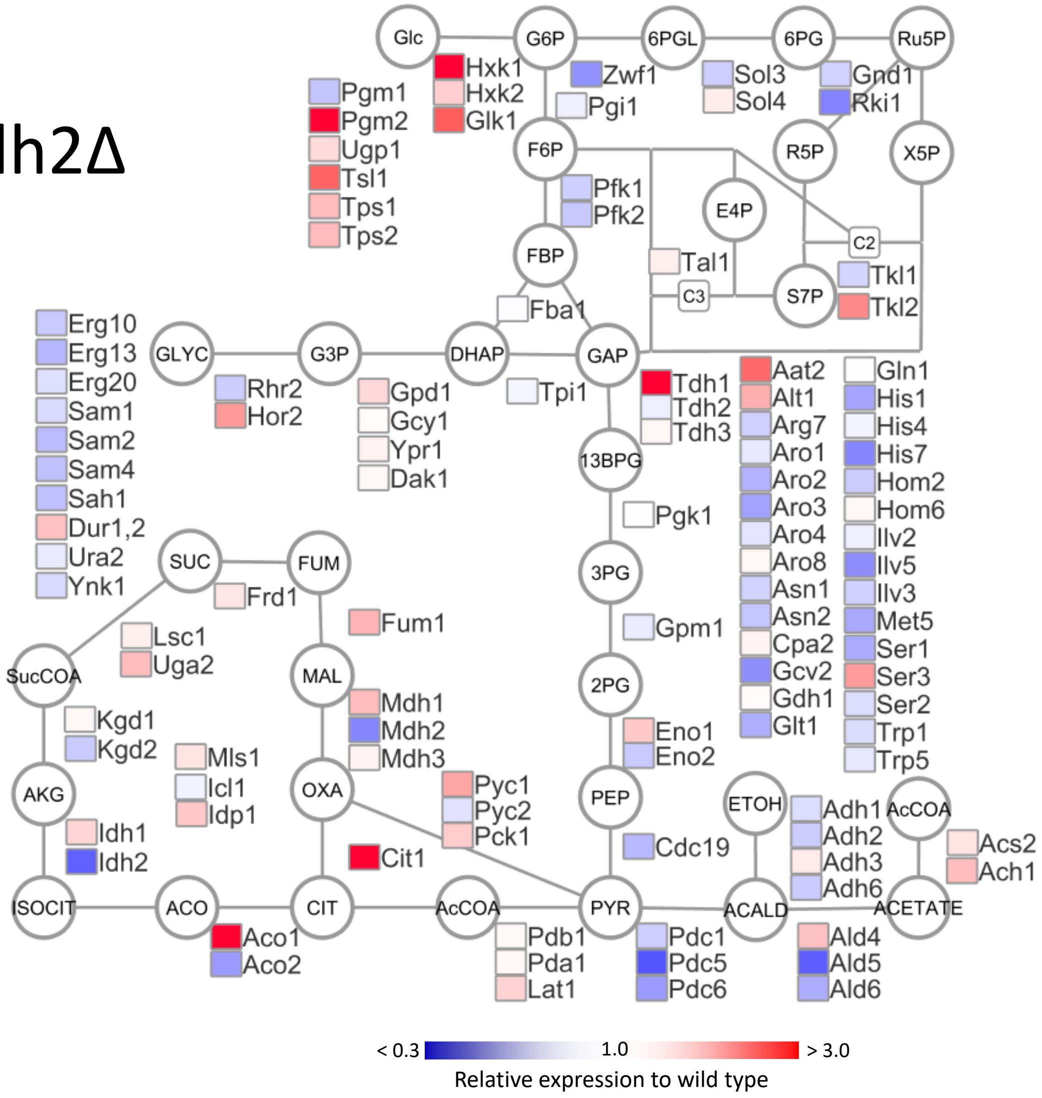

Glc
G6P
6PGL
6PG
Ru5P
Hxk1
Hxk2
Glk1
Zwf1
Pgi1
Sol3
Sol4
Gnd1
Rki1
Pgm1
Pgm2
Ugp1
Tsl1
Tps1
Tps2
F6P
R5P
X5P
Pfk1
Pfk2
E4P
C2
FBP
Tal1
C3
S7P
Tkl1
Tkl2
Fba1
Erg10
Erg13
Erg20
Sam1
Sam2
Sam4
Sah1
Dur1,2
Ura2
Ynk1
GLYC
G3P
DHAP
GAP
Rhr2
Hor2
Gpd1
Gcy1
Ypr1
Dak1
Tpi1
Tdh1
Tdh2
Tdh3
13BPG
Pgk1
3PG
Gpm1
2PG
Eno1
Eno2
PEP
Cdc19
PYR
Aat2
Alt1
Arg7
Aro1
Aro2
Aro3
Aro4
Aro8
Asn1
Asn2
Cpa2
Gcv2
Gdh1
Glt1
Gln1
His1
His4
His7
Hom2
Hom6
Ilv2
Ilv5
Ilv3
Met5
Ser1
Ser3
Ser2
Trp1
Trp5
SUC
FUM
Frd1
Lsc1
Uga2
Fum1
SucCOA
MAL
Mdh1
Mdh2
Mdh3
Kgd1
Kgd2
Mls1
Icl1
Idp1
AKG
OXA
Pyc1
Pyc2
Pck1
Idh1
Idh2
Cit1
ISOCIT
ACO
CIT
AcCOA
Aco1
Aco2
Pdb1
Pda1
Lat1
Pdc1
Pdc5
Pdc6
ETOH
Adh1
Adh2
Adh3
Adh6
AcCOA
ACALD
ACETATE
Acs2
Ach1
Ald4
Ald5
Ald6
< 0.3
1.0
> 3.0
Relative expression to wild type

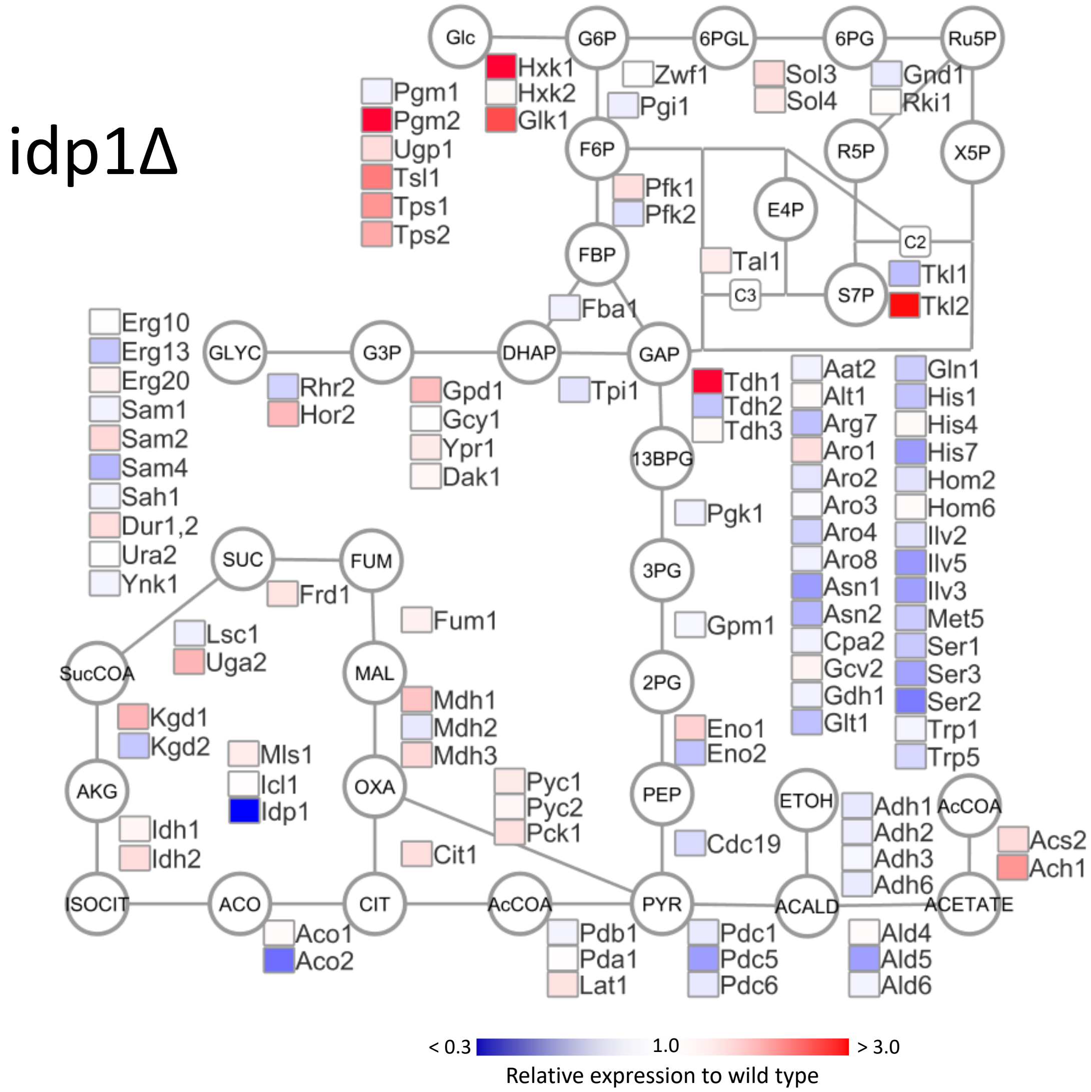
idp1Δ
Glc
G6P
6PGL
6PG
Ru5P
Hxk1
Hxk2
Glk1
Zwf1
Pgi1
Sol3
Sol4
Gnd1
Rki1
Pgm1
Pgm2
Ugp1
Tsl1
Tps1
Tps2
F6P
R5P
X5P
Pfk1
Pfk2
E4P
C2
FBP
Tal1
C3
S7P
Tkl1
Tkl2
Fba1
Erg10
Erg13
Erg20
Sam1
Sam2
Sam4
Sah1
Dur1,2
Ura2
Ynk1
GLYC
G3P
DHAP
GAP
Rhr2
Hor2
Gpd1
Gcy1
Ypr1
Dak1
Tpi1
Tdh1
Tdh2
Tdh3
Aat2
Alt1
Arg7
Aro1
Aro2
Aro3
Aro4
Aro8
Asn1
Asn2
Cpa2
Gcv2
Gdh1
Glt1
Gln1
His1
His4
His7
Hom2
Hom6
Ilv2
Ilv5
Ilv3
Met5
Ser1
Ser3
Ser2
Trp1
Trp5
13BPG
Pgk1
3PG
Gpm1
2PG
Eno1
Eno2
PEP
Cdc19
SUC
FUM
Frd1
Fum1
Lsc1
Uga2
SucCOA
MAL
Mdh1
Mdh2
Mdh3
Kgd1
Kgd2
Mls1
Icl1
Idp1
AKG
OXA
Pyc1
Pyc2
Pck1
Idh1
Idh2
Cit1
ETOH
Adh1
Adh2
Adh3
Adh6
AcCOA
Acs2
Ach1
ISOCIT
ACO
CIT
AcCOA
PYR
ACALD
ACETATE
Aco1
Aco2
Pdb1
Pda1
Lat1
Pdc1
Pdc5
Pdc6
Ald4
Ald5
Ald6
< 0.3
1.0
> 3.0
Relative expression to wild type

lpd1Δ

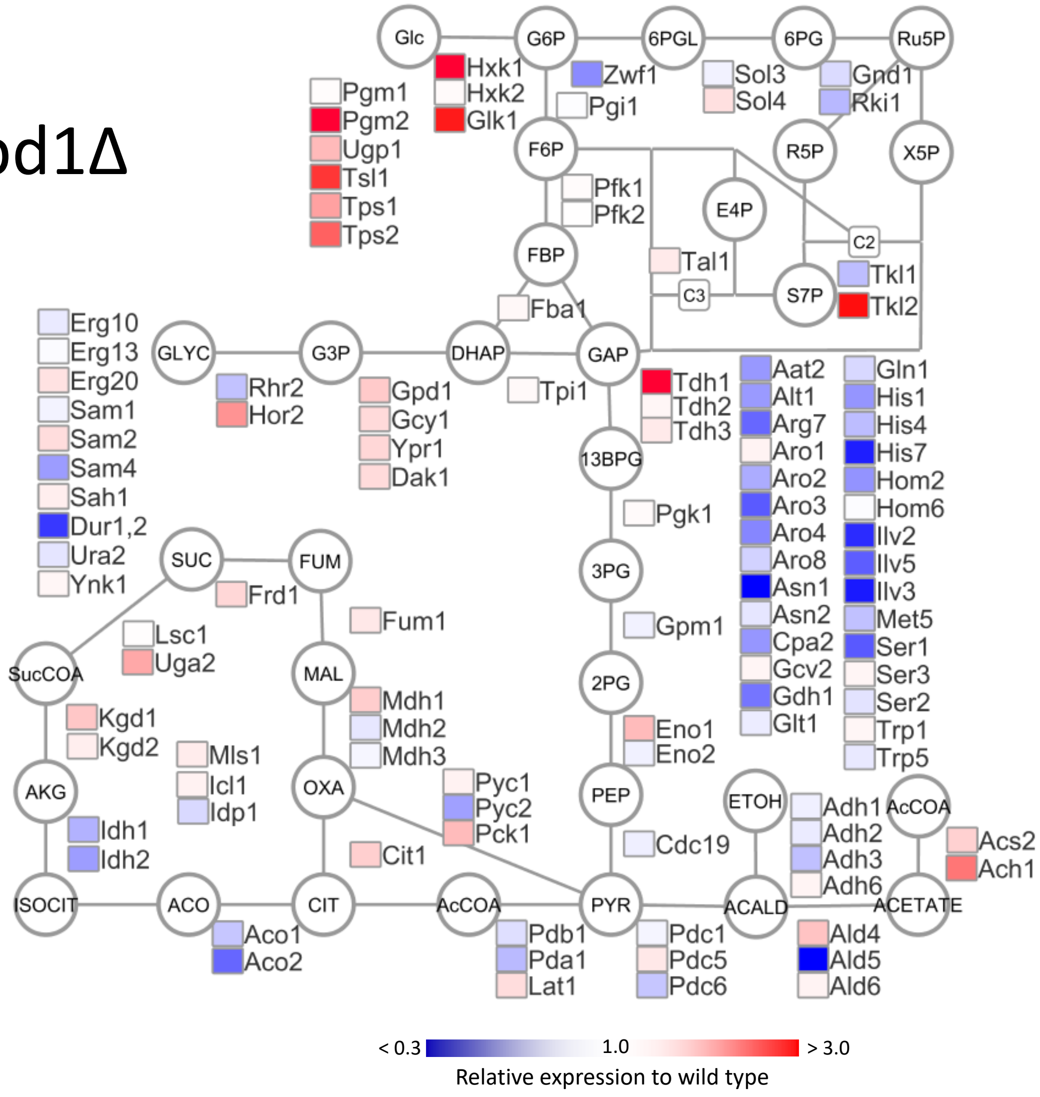
Glc
G6P
6PGL
6PG
Ru5P
Hxk1
Hxk2
Glk1
Zwf1
Pgi1
Sol3
Sol4
Gnd1
Rki1
Pgm1
Pgm2
Ugp1
Tsl1
Tps1
Tps2
F6P
R5P
X5P
Pfk1
Pfk2
E4P
C2
FBP
Tal1
C3
S7P
Tkl1
Tkl2
Fba1
Erg10
Erg13
Erg20
Sam1
Sam2
Sam4
Sah1
Dur1,2
Ura2
Ynk1
GLYC
G3P
DHAP
GAP
Rhr2
Hor2
Gpd1
Gcy1
Ypr1
Dak1
Tpi1
Tdh1
Tdh2
Tdh3
13BPG
Pgk1
3PG
Gpm1
2PG
Eno1
Eno2
PEP
Cdc19
Aat2
Alt1
Arg7
Aro1
Aro2
Aro3
Aro4
Aro8
Asn1
Asn2
Cpa2
Gcv2
Gdh1
Glt1
Gln1
His1
His4
His7
Hom2
Hom6
Ilv2
Ilv5
Ilv3
Met5
Ser1
Ser3
Ser2
Trp1
Trp5
SUC
FUM
Frd1
Fum1
Lsc1
Uga2
SucCOA
MAL
Mdh1
Mdh2
Mdh3
Kgd1
Kgd2
Mls1
Icl1
Idp1
AKG
OXA
Pyc1
Pyc2
Pck1
Idh1
Idh2
Cit1
ETOH
Adh1
Adh2
Adh3
Adh6
AcCOA
Acs2
Ach1
ISOCIT
ACO
CIT
AcCOA
PYR
ACALD
ACETATE
Aco1
Aco2
Pdb1
Pda1
Lat1
Pdc1
Pdc5
Pdc6
Ald4
Ald5
Ald6
< 0.3
1.0
> 3.0
Relative expression to wild type

mae1Δ

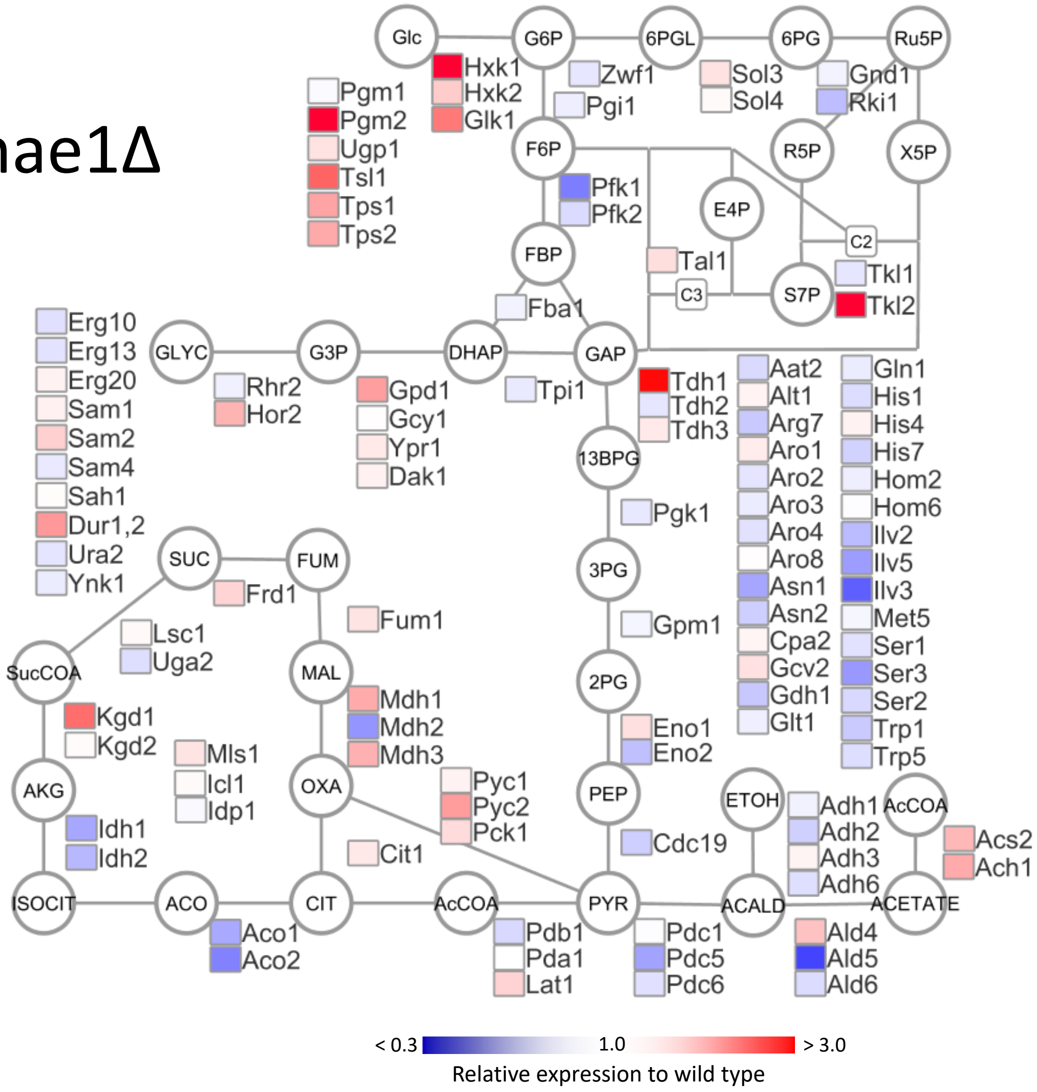

Glc
G6P
6PGL
6PG
Ru5P
Hxk1
Hxk2
Glk1
Zwf1
Pgi1
Sol3
Sol4
Gnd1
Rki1
Pgm1
Pgm2
Ugp1
Tsl1
Tps1
Tps2
F6P
R5P
X5P
Pfk1
Pfk2
E4P
C2
FBP
Tal1
C3
S7P
Tkl1
Tkl2
Fba1
Erg10
Erg13
Erg20
Sam1
Sam2
Sam4
Sah1
Dur1,2
Ura2
Ynk1
GLYC
G3P
DHAP
GAP
Rhr2
Hor2
Gpd1
Gcy1
Ypr1
Dak1
Tpi1
Tdh1
Tdh2
Tdh3
13BPG
Aat2
Alt1
Arg7
Aro1
Aro2
Aro3
Aro4
Aro8
Asn1
Asn2
Cpa2
Gcv2
Gdh1
Glt1
Gln1
His1
His4
His7
Hom2
Hom6
Ilv2
Ilv5
Ilv3
Met5
Ser1
Ser3
Ser2
Trp1
Trp5
Pgk1
3PG
SUC
FUM
Frd1
Fum1
Gpm1
2PG
SucCOA
Lsc1
Uga2
MAL
Kgd1
Kgd2
Mdh1
Mdh2
Mdh3
Eno1
Eno2
AKG
Mls1
Icl1
Idp1
OXA
Pyc1
Pyc2
Pck1
PEP
ETOH
Adh1
Adh2
Adh3
Adh6
AcCOA
Acs2
Ach1
Idh1
Idh2
Cit1
Cdc19
ISOCIT
ACO
CIT
AcCOA
PYR
ACALD
ACETATE
Aco1
Aco2
Pdb1
Pda1
Lat1
Pdc1
Pdc5
Pdc6
Ald4
Ald5
Ald6
< 0.3
1.0
> 3.0
Relative expression to wild type

mdh1Δ

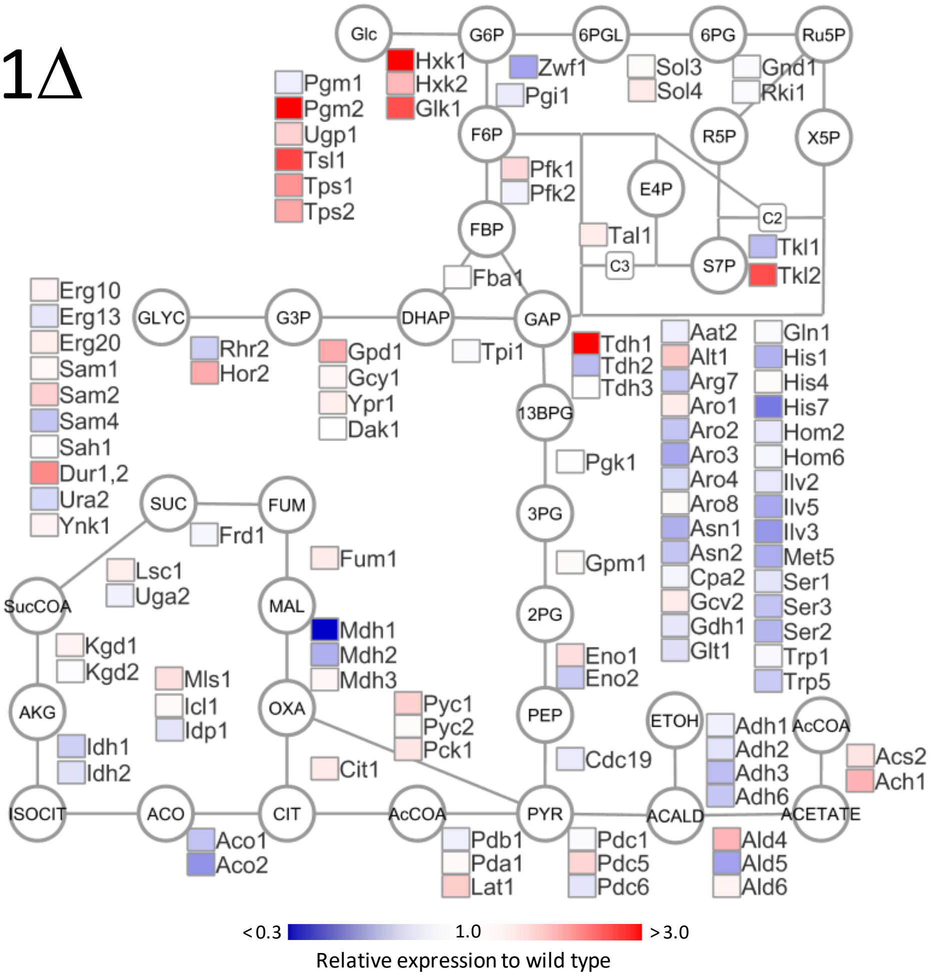

Glc
G6P
6PGL
6PG
Ru5P
Hxk1
Hxk2
Glk1
Zwf1
Pgi1
Sol3
Sol4
Gnd1
Rki1
Pgm1
Pgm2
Ugp1
Tsl1
Tps1
Tps2
F6P
R5P
X5P
Pfk1
Pfk2
E4P
FBP
C2
Tal1
C3
S7P
Tkl1
Tkl2
Fba1
Erg10
Erg13
Erg20
Sam1
Sam2
Sam4
Sah1
Dur1,2
Ura2
Ynk1
GLYC
G3P
DHAP
GAP
Rhr2
Hor2
Gpd1
Gcy1
Ypr1
Dak1
Tpi1
Tdh1
Tdh2
Tdh3
13BPG
Pgk1
3PG
Gpm1
2PG
Eno1
Eno2
PEP
Cdc19
Aat2
Alt1
Arg7
Aro1
Aro2
Aro3
Aro4
Aro8
Asn1
Asn2
Cpa2
Gcv2
Gdh1
Glt1
Gln1
His1
His4
His7
Hom2
Hom6
Ilv2
Ilv5
Ilv3
Met5
Ser1
Ser3
Ser2
Trp1
Trp5
SUC
FUM
Frd1
Fum1
Lsc1
Uga2
SucCOA
MAL
Mdh1
Mdh2
Mdh3
Kgd1
Kgd2
Mls1
Icl1
Idp1
AKG
OXA
Pyc1
Pyc2
Pck1
Idh1
Idh2
Cit1
ETOH
Adh1
Adh2
Adh3
Adh6
AcCOA
Acs2
Ach1
ISOCIT
ACO
CIT
AcCOA
PYR
ACALD
ACETATE
Aco1
Aco2
Pdb1
Pda1
Lat1
Pdc1
Pdc5
Pdc6
Ald4
Ald5
Ald6
< 0.3
1.0
> 3.0
Relative expression to wild type

mdh2Δ

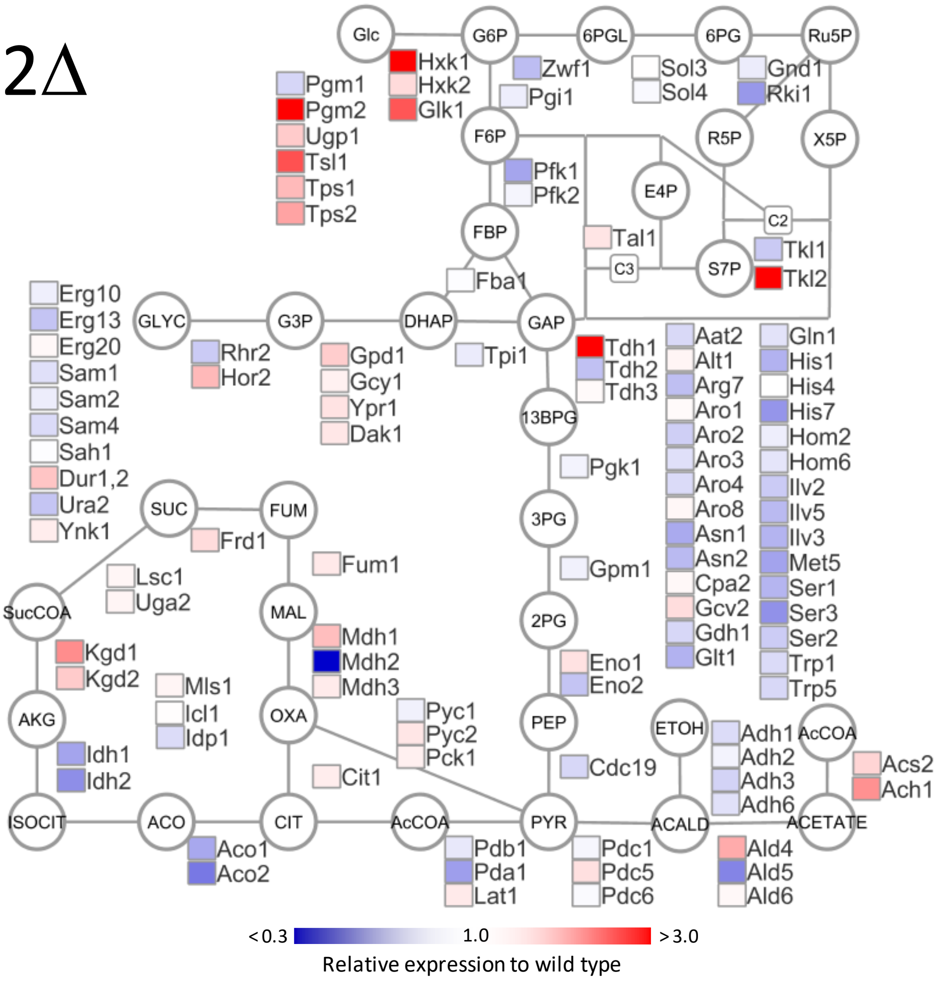

Glc
G6P
6PGL
6PG
Ru5P
Hxk1
Hxk2
Glk1
Zwf1
Pgi1
Sol3
Sol4
Gnd1
Rki1
Pgm1
Pgm2
Ugp1
Tsl1
Tps1
Tps2
F6P
R5P
X5P
Pfk1
Pfk2
E4P
FBP
C2
Tal1
C3
S7P
Tkl1
Tkl2
Fba1
Erg10
Erg13
Erg20
Sam1
Sam2
Sam4
Sah1
Dur1,2
Ura2
Ynk1
GLYC
G3P
DHAP
GAP
Rhr2
Hor2
Gpd1
Gcy1
Ypr1
Dak1
Tpi1
Tdh1
Tdh2
Tdh3
13BPG
Aat2
Alt1
Arg7
Aro1
Aro2
Aro3
Aro4
Aro8
Asn1
Asn2
Cpa2
Gcv2
Gdh1
Glt1
Gln1
His1
His4
His7
Hom2
Hom6
Ilv2
Ilv5
Ilv3
Met5
Ser1
Ser3
Ser2
Trp1
Trp5
SUC
FUM
Frd1
Fum1
Pgk1
3PG
Gpm1
2PG
SucCOA
Lsc1
Uga2
MAL
Mdh1
Mdh2
Mdh3
Kgd1
Kgd2
Mls1
Icl1
Idp1
Eno1
Eno2
AKG
OXA
Pyc1
Pyc2
Pck1
PEP
ETOH
Adh1
Adh2
Adh3
Adh6
AcCOA
Acs2
Ach1
Idh1
Idh2
Cit1
Cdc19
ISOCIT
ACO
CIT
AcCOA
PYR
ACALD
ACETATE
Aco1
Aco2
Pdb1
Pda1
Lat1
Pdc1
Pdc5
Pdc6
Ald4
Ald5
Ald6
<0.3
1.0
>3.0
Relative expression to wild type

pda1Δ

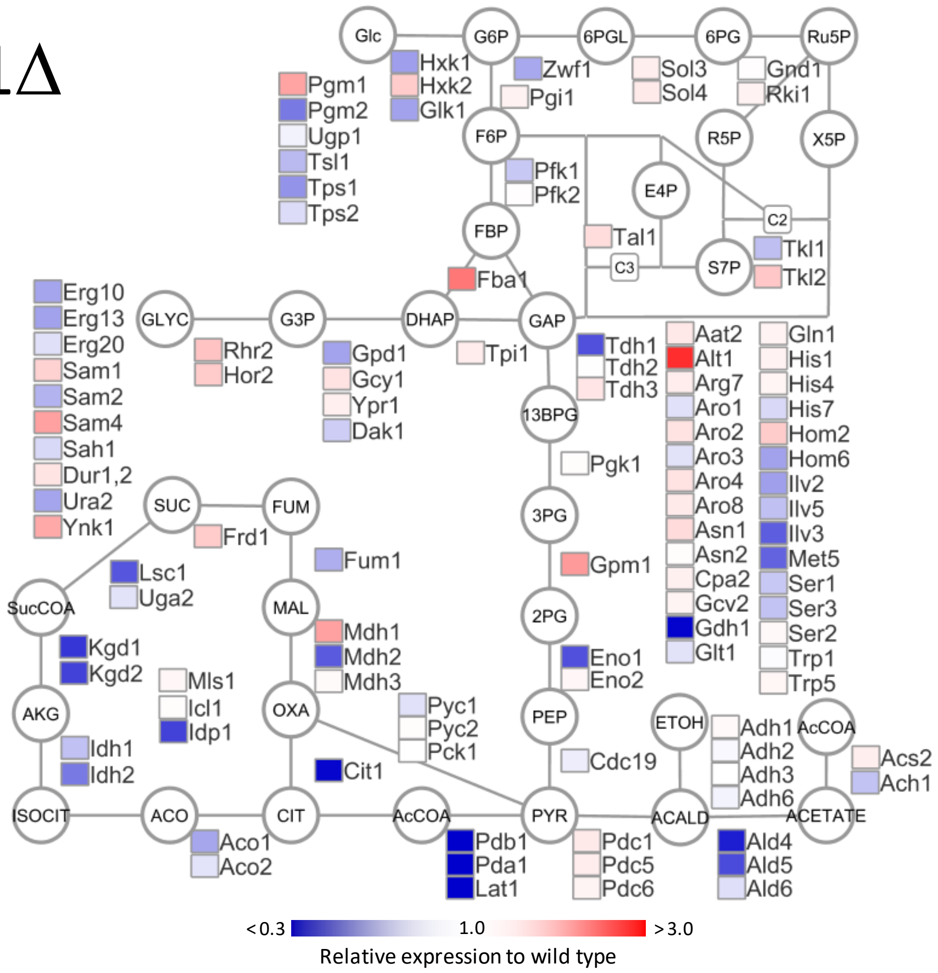
Glc
G6P
6PGL
6PG
Ru5P
Hxk1
Hxk2
Glk1
Zwf1
Pgi1
Sol3
Sol4
Gnd1
Rki1
Pgm1
Pgm2
Ugp1
Tsl1
Tps1
Tps2
F6P
R5P
X5P
Pfk1
Pfk2
E4P
C2
FBP
Tal1
C3
S7P
Tkl1
Tkl2
Fba1
Erg10
Erg13
Erg20
Sam1
Sam2
Sam4
Sah1
Dur1,2
Ura2
Ynk1
GLYC
G3P
DHAP
GAP
Rhr2
Hor2
Gpd1
Gcy1
Ypr1
Dak1
Tpi1
Tdh1
Tdh2
Tdh3
13BPG
Pgk1
3PG
Gpm1
2PG
Eno1
Eno2
PEP
Cdc19
Aat2
Alt1
Arg7
Aro1
Aro2
Aro3
Aro4
Aro8
Asn1
Asn2
Cpa2
Gcv2
Gdh1
Glt1
Gln1
His1
His4
His7
Hom2
Hom6
Ilv2
Ilv5
Ilv3
Met5
Ser1
Ser3
Ser2
Trp1
Trp5
SUC
FUM
Frd1
Fum1
Lsc1
Uga2
SucCOA
MAL
Mdh1
Mdh2
Mdh3
Kgd1
Kgd2
Mls1
Icl1
Idp1
AKG
OXA
Pyc1
Pyc2
Pck1
Idh1
Idh2
Cit1
ISOCIT
ACO
CIT
AcCOA
PYR
ACALD
ACETATE
Aco1
Aco2
Pdb1
Pda1
Lat1
Pdc1
Pdc5
Pdc6
ETOH
Adh1
Adh2
Adh3
Adh6
AcCOA
Acs2
Ach1
Ald4
Ald5
Ald6
<0.3
1.0
>3.0
Relative expression to wild type

pdc1Δ

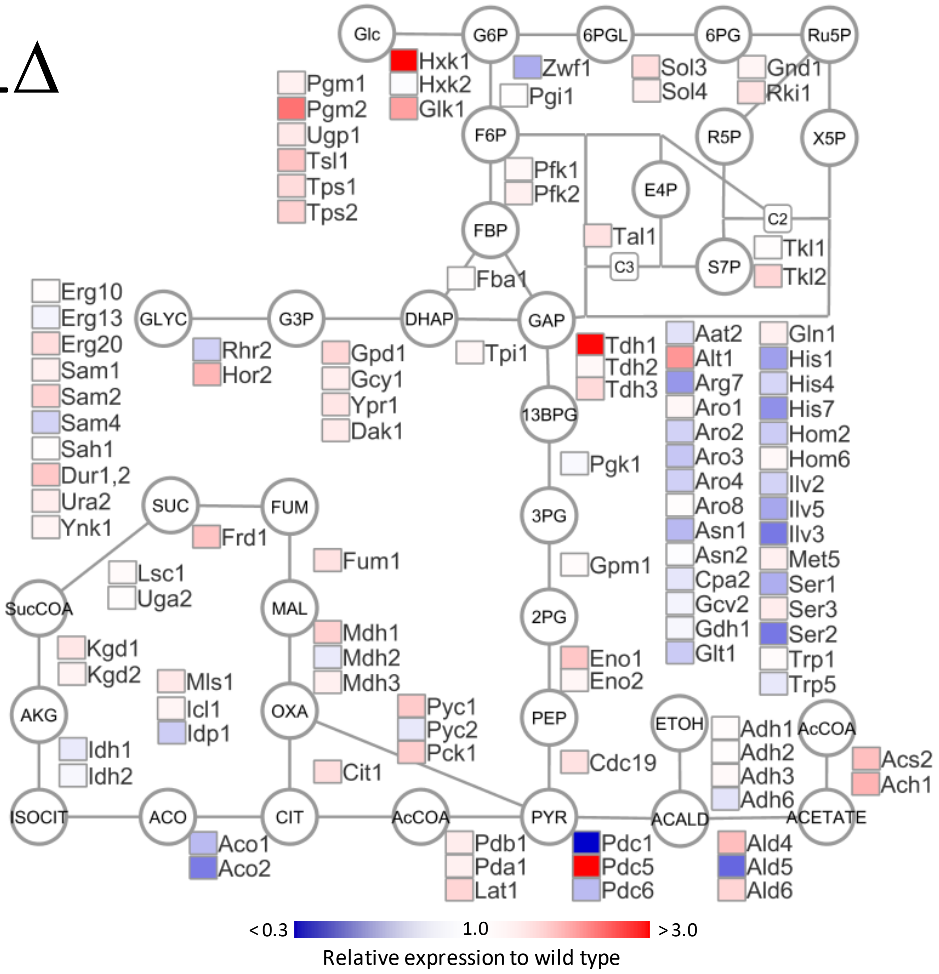
Glc
G6P
6PGL
6PG
Ru5P
Hxk1
Hxk2
Glk1
Zwf1
Pgi1
Sol3
Sol4
Gnd1
Rki1
Pgm1
Pgm2
Ugp1
Tsl1
Tps1
Tps2
F6P
R5P
X5P
Pfk1
Pfk2
E4P
C2
FBP
Tal1
C3
S7P
Tkl1
Tkl2
Fba1
Erg10
Erg13
Erg20
Sam1
Sam2
Sam4
Sah1
Dur1,2
Ura2
Ynk1
GLYC
G3P
DHAP
GAP
Rhr2
Hor2
Gpd1
Gcy1
Ypr1
Dak1
Tpi1
Tdh1
Tdh2
Tdh3
13BPG
Aat2
Alt1
Arg7
Aro1
Aro2
Aro3
Aro4
Aro8
Asn1
Asn2
Cpa2
Gcv2
Gdh1
Glt1
Gln1
His1
His4
His7
Hom2
Hom6
Ilv2
Ilv5
Ilv3
Met5
Ser1
Ser3
Ser2
Trp1
Trp5
SUC
FUM
Frd1
Fum1
Pgk1
3PG
Gpm1
2PG
SucCOA
Lsc1
Uga2
MAL
Mdh1
Mdh2
Mdh3
Kgd1
Kgd2
Mls1
Icl1
Idp1
AKG
OXA
Pyc1
Pyc2
Pck1
Eno1
Eno2
PEP
ETOH
Adh1
Adh2
Adh3
Adh6
AcCOA
Acs2
Ach1
Idh1
Idh2
Cdc19
Cit1
ISOCIT
ACO
CIT
AcCOA
PYR
ACALD
ACETATE
Aco1
Aco2
Pdb1
Pda1
Lat1
Pdc1
Pdc5
Pdc6
Ald4
Ald5
Ald6
< 0.3
1.0
> 3.0
Relative expression to wild type

pfk1Δ

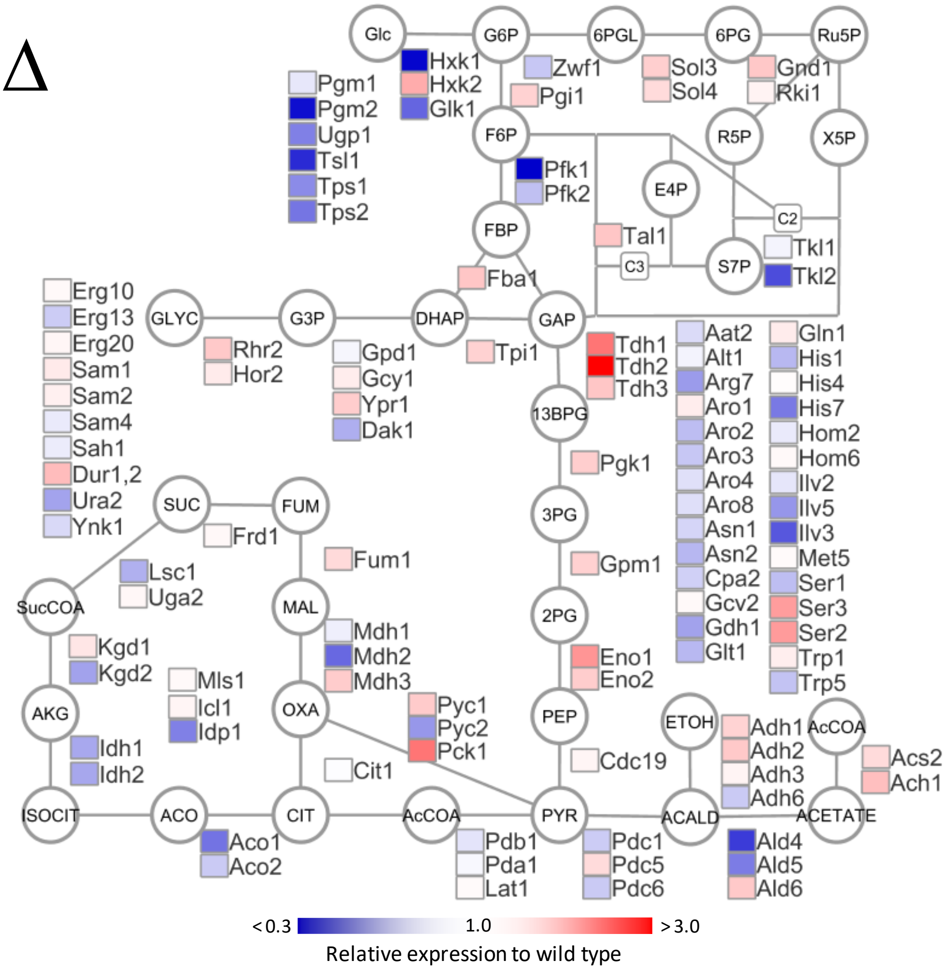
Glc
G6P
6PGL
6PG
Ru5P
Hxk1
Hxk2
Glk1
Zwf1
Pgi1
Sol3
Sol4
Gnd1
Rki1
Pgm1
Pgm2
Ugp1
Tsl1
Tps1
Tps2
F6P
R5P
X5P
Pfk1
Pfk2
E4P
FBP
C2
Tal1
Tkl1
Tkl2
C3
S7P
Fba1
Erg10
Erg13
Erg20
Sam1
Sam2
Sam4
Sah1
Dur1,2
Ura2
Ynk1
GLYC
G3P
DHAP
GAP
Rhr2
Hor2
Gpd1
Gcy1
Ypr1
Dak1
Tpi1
Tdh1
Tdh2
Tdh3
13BPG
Pgk1
Aat2
Alt1
Arg7
Aro1
Aro2
Aro3
Aro4
Aro8
Asn1
Asn2
Cpa2
Gcv2
Gdh1
Glt1
Gln1
His1
His4
His7
Hom2
Hom6
Ilv2
Ilv5
Ilv3
Met5
Ser1
Ser3
Ser2
Trp1
Trp5
SUC
FUM
Frd1
Fum1
3PG
Gpm1
SucCOA
Lsc1
Uga2
MAL
2PG
Mdh1
Mdh2
Mdh3
Kgd1
Kgd2
Mls1
Icl1
Idp1
Eno1
Eno2
AKG
OXA
Pyc1
Pyc2
Pck1
PEP
ETOH
AcCOA
Adh1
Adh2
Adh3
Adh6
Acs2
Ach1
Idh1
Idh2
Cit1
Cdc19
ISOCIT
ACO
CIT
AcCOA
PYR
ACALD
ACETATE
Aco1
Aco2
Pdb1
Pda1
Lat1
Pdc1
Pdc5
Pdc6
Ald4
Ald5
Ald6
<0.3
1.0
>3.0
Relative expression to wild type

pyc2Δ

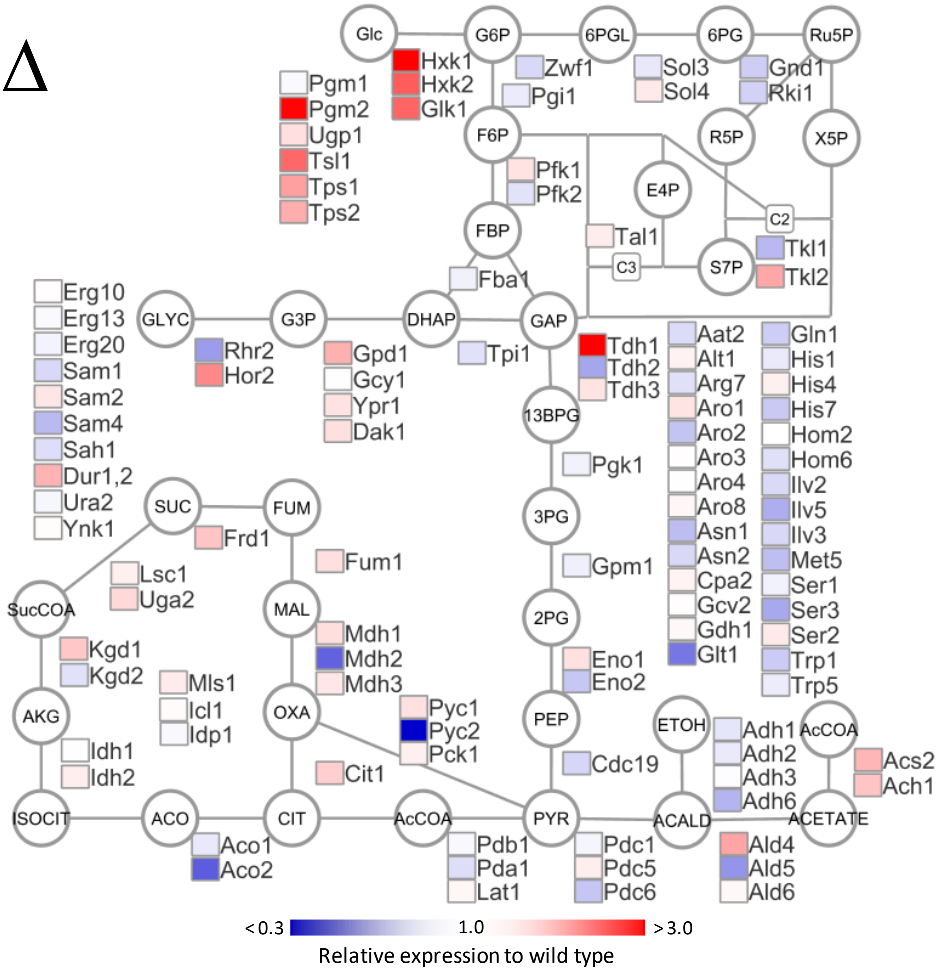

Glc
G6P
6PGL
6PG
Ru5P
Hxk1
Hxk2
Glk1
Zwf1
Pgi1
Sol3
Sol4
Gnd1
Rki1
Pgm1
Pgm2
Ugp1
Tsl1
Tps1
Tps2
F6P
R5P
X5P
Pfk1
Pfk2
E4P
FBP
C2
Tal1
C3
S7P
Tkl1
Tkl2
Fba1
Erg10
Erg13
Erg20
Sam1
Sam2
Sam4
Sah1
Dur1,2
Ura2
Ynk1
GLYC
G3P
DHAP
GAP
Rhr2
Hor2
Gpd1
Gcy1
Ypr1
Dak1
Tpi1
Tdh1
Tdh2
Tdh3
13BPG
Aat2
Alt1
Arg7
Aro1
Aro2
Aro3
Aro4
Aro8
Asn1
Asn2
Cpa2
Gcv2
Gdh1
Glt1
Gln1
His1
His4
His7
Hom2
Hom6
Ilv2
Ilv5
Ilv3
Met5
Ser1
Ser3
Ser2
Trp1
Trp5
Pgk1
SUC
FUM
Frd1
3PG
Fum1
Lsc1
Uga2
SucCOA
MAL
Gpm1
2PG
Kgd1
Kgd2
Mdh1
Mdh2
Mdh3
Mls1
Icl1
Idp1
Eno1
Eno2
AKG
OXA
Pyc1
Pyc2
Pck1
PEP
ETOH
Adh1
Adh2
Adh3
Adh6
AcCOA
Acs2
Ach1
Idh1
Idh2
Cit1
Cdc19
ISOCIT
ACO
CIT
AcCOA
PYR
ACALD
ACETATE
Aco1
Aco2
Pdb1
Pda1
Lat1
Pdc1
Pdc5
Pdc6
Ald4
Ald5
Ald6
< 0.3
1.0
> 3.0
Relative expression to wild type

rpe1Δ

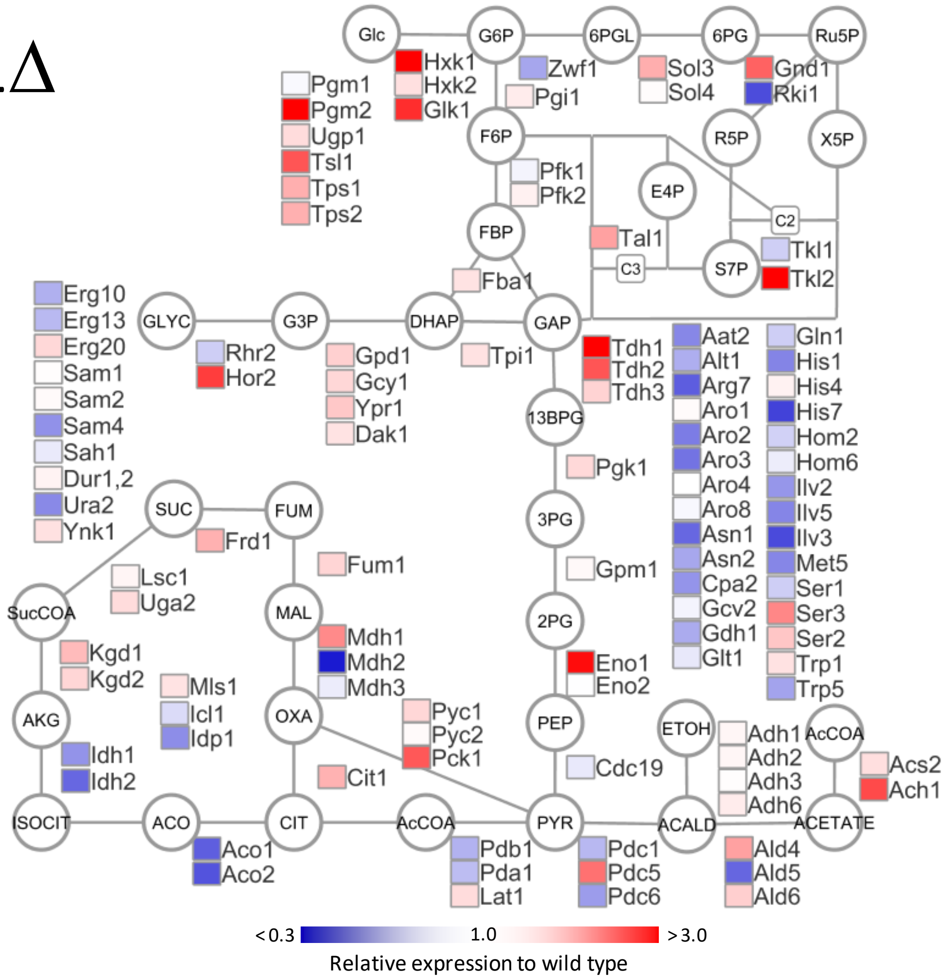

Glc
G6P
6PGL
6PG
Ru5P
Hxk1
Hxk2
Glk1
Zwf1
Pgi1
Sol3
Sol4
Gnd1
Rki1
Pgm1
Pgm2
Ugp1
Tsl1
Tps1
Tps2
F6P
R5P
X5P
Pfk1
Pfk2
E4P
FBP
C2
Tal1
C3
S7P
Tkl1
Tkl2
Fba1
Erg10
Erg13
Erg20
Sam1
Sam2
Sam4
Sah1
Dur1,2
Ura2
Ynk1
GLYC
G3P
DHAP
GAP
Rhr2
Hor2
Gpd1
Gcy1
Ypr1
Dak1
Tpi1
Tdh1
Tdh2
Tdh3
13BPG
Pgk1
3PG
Gpm1
2PG
Eno1
Eno2
PEP
Cdc19
Aat2
Alt1
Arg7
Aro1
Aro2
Aro3
Aro4
Aro8
Asn1
Asn2
Cpa2
Gcv2
Gdh1
Glt1
Gln1
His1
His4
His7
Hom2
Hom6
Ilv2
Ilv5
Ilv3
Met5
Ser1
Ser3
Ser2
Trp1
Trp5
SUC
FUM
Frd1
Lsc1
Uga2
SucCOA
MAL
Fum1
Kgd1
Kgd2
Mdh1
Mdh2
Mdh3
Mls1
Icl1
Idp1
AKG
OXA
Pyc1
Pyc2
Pck1
Idh1
Idh2
Cit1
ISOCIT
ACO
CIT
AcCOA
PYR
Aco1
Aco2
Pdb1
Pda1
Lat1
Pdc1
Pdc5
Pdc6
ETOH
Adh1
Adh2
Adh3
Adh6
AcCOA
Acs2
Ach1
ACALD
ACETATE
Ald4
Ald5
Ald6
<0.3
1.0
>3.0
Relative expression to wild type

tal1Δ

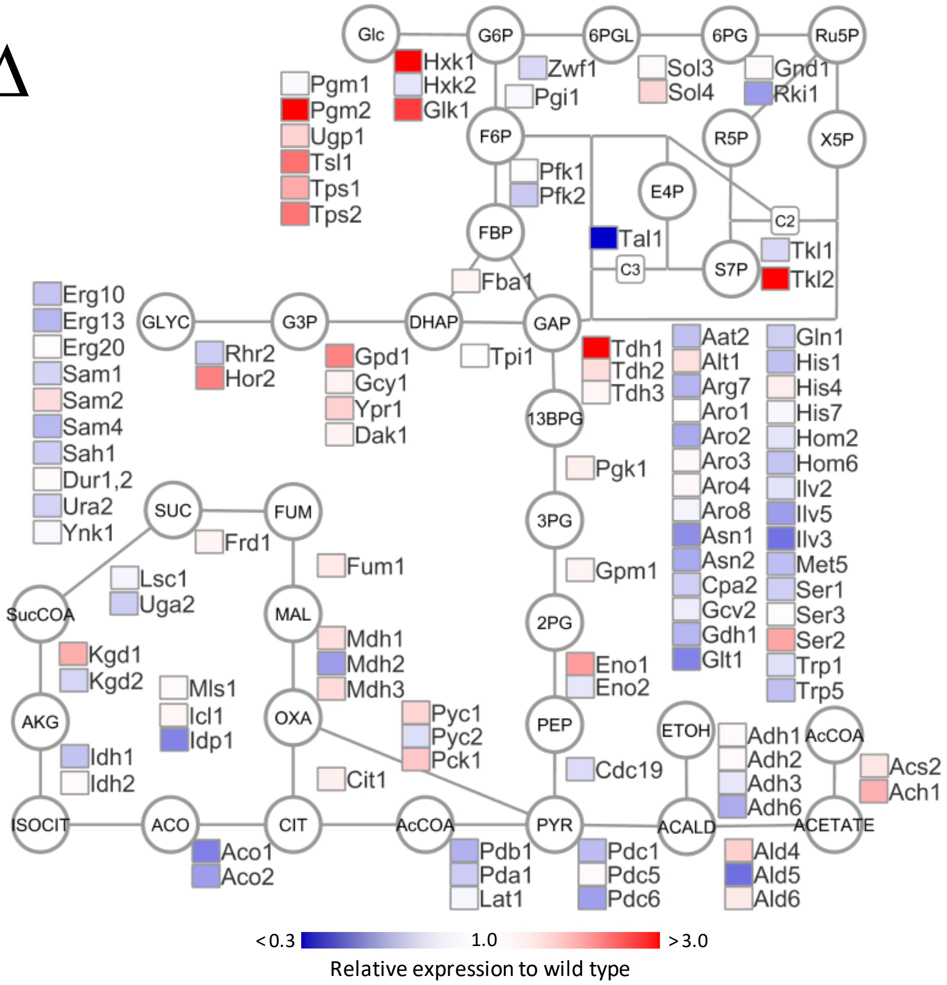

Glc
G6P
6PGL
6PG
Ru5P
Hxk1
Hxk2
Glk1
Pgm1
Pgm2
Ugp1
Tsl1
Tps1
Tps2
Zwf1
Pgi1
Sol3
Sol4
Gnd1
Rki1
F6P
R5P
X5P
Pfk1
Pfk2
E4P
C2
FBP
Tal1
C3
S7P
Tkl1
Tkl2
Fba1
Erg10
Erg13
Erg20
Sam1
Sam2
Sam4
Sah1
Dur1,2
Ura2
Ynk1
GLYC
G3P
DHAP
GAP
Rhr2
Hor2
Gpd1
Gcy1
Ypr1
Dak1
Tpi1
Tdh1
Tdh2
Tdh3
13BPG
Aat2
Alt1
Arg7
Aro1
Aro2
Aro3
Aro4
Aro8
Asn1
Asn2
Cpa2
Gcv2
Gdh1
Glt1
Gln1
His1
His4
His7
Hom2
Hom6
Ilv2
Ilv5
Ilv3
Met5
Ser1
Ser3
Ser2
Trp1
Trp5
Pgk1
3PG
Gpm1
2PG
Eno1
Eno2
PEP
Cdc19
SUC
FUM
Frd1
Fum1
Lsc1
Uga2
SucCOA
MAL
Mdh1
Mdh2
Mdh3
Kgd1
Kgd2
Mls1
Icl1
Idp1
AKG
OXA
Pyc1
Pyc2
Pck1
Idh1
Idh2
Cit1
ETOH
Adh1
Adh2
Adh3
Adh6
AcCOA
Acs2
Ach1
ISOCIT
ACO
CIT
AcCOA
PYR
ACALD
ACETATE
Aco1
Aco2
Pdb1
Pda1
Lat1
Pdc1
Pdc5
Pdc6
Ald4
Ald5
Ald6
< 0.3
1.0
> 3.0
Relative expression to wild type

tdh1Δ

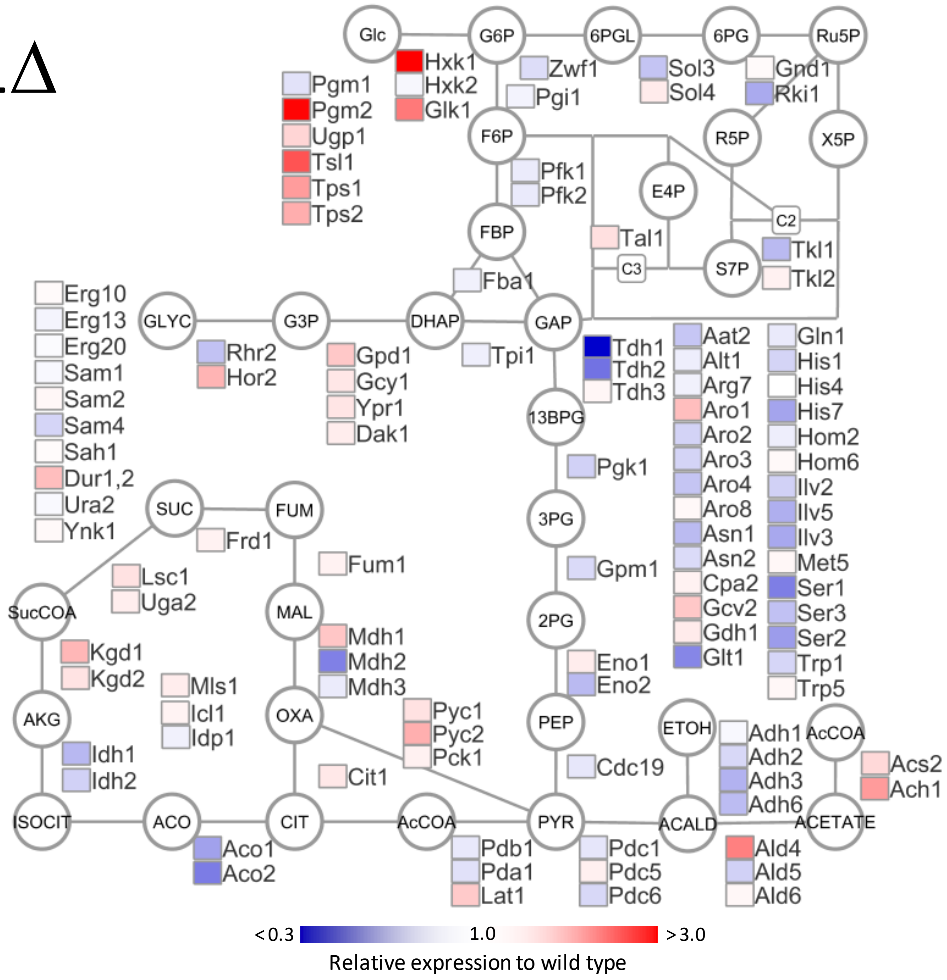

Glc
G6P
6PGL
6PG
Ru5P
Hxk1
Hxk2
Glk1
Zwf1
Pgi1
Sol3
Sol4
Gnd1
Rki1
Pgm1
Pgm2
Ugp1
Tsl1
Tps1
Tps2
F6P
R5P
X5P
Pfk1
Pfk2
E4P
C2
FBP
Tal1
C3
S7P
Tkl1
Tkl2
Fba1
Erg10
Erg13
Erg20
Sam1
Sam2
Sam4
Sah1
Dur1,2
Ura2
Ynk1
GLYC
G3P
DHAP
GAP
Rhr2
Hor2
Gpd1
Gcy1
Ypr1
Dak1
Tpi1
Tdh1
Tdh2
Tdh3
13BPG
Aat2
Alt1
Arg7
Aro1
Aro2
Aro3
Aro4
Aro8
Asn1
Asn2
Cpa2
Gcv2
Gdh1
Glt1
Gln1
His1
His4
His7
Hom2
Hom6
Ilv2
Ilv5
Ilv3
Met5
Ser1
Ser3
Ser2
Trp1
Trp5
Pgk1
3PG
Gpm1
2PG
Eno1
Eno2
PEP
Cdc19
SUC
FUM
Frd1
Fum1
Lsc1
Uga2
SucCOA
MAL
Mdh1
Mdh2
Mdh3
Kgd1
Kgd2
Mls1
Icl1
Idp1
AKG
OXA
Pyc1
Pyc2
Pck1
Idh1
Idh2
Cit1
ETOH
Adh1
Adh2
Adh3
Adh6
AcCOA
Acs2
Ach1
ISOCIT
ACO
CIT
AcCOA
PYR
ACALD
ACETATE
Aco1
Aco2
Pdb1
Pda1
Lat1
Pdc1
Pdc5
Pdc6
Ald4
Ald5
Ald6
<0.3
1.0
>3.0
Relative expression to wild type

tdh2Δ

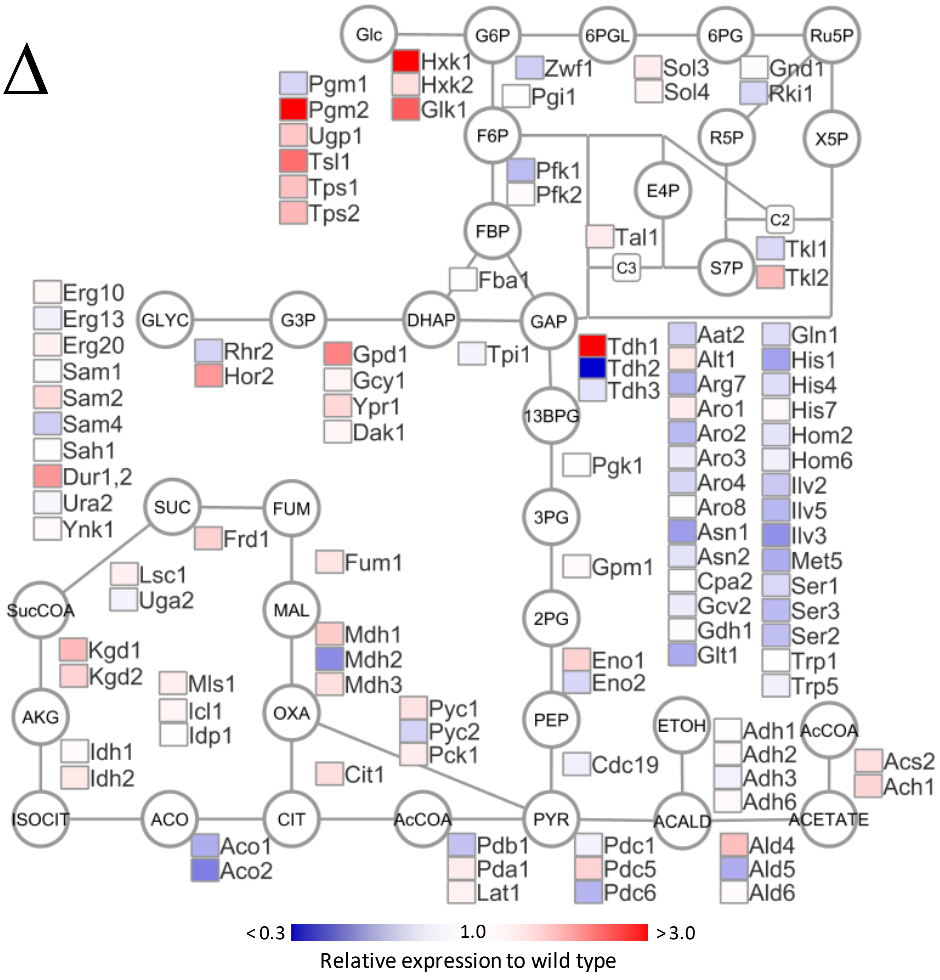

Glc
G6P
6PGL
6PG
Ru5P
Hxk1
Hxk2
Glk1
Zwf1
Pgi1
Sol3
Sol4
Gnd1
Rki1
Pgm1
Pgm2
Ugp1
Tsl1
Tps1
Tps2
F6P
R5P
X5P
Pfk1
Pfk2
E4P
C2
FBP
Tal1
C3
S7P
Tkl1
Tkl2
Fba1
Erg10
Erg13
Erg20
Sam1
Sam2
Sam4
Sah1
Dur1,2
Ura2
Ynk1
GLYC
G3P
DHAP
GAP
Rhr2
Hor2
Gpd1
Gcy1
Ypr1
Dak1
Tpi1
Tdh1
Tdh2
Tdh3
13BPG
Pgk1
3PG
Gpm1
2PG
Eno1
Eno2
PEP
Cdc19
Aat2
Alt1
Arg7
Aro1
Aro2
Aro3
Aro4
Aro8
Asn1
Asn2
Cpa2
Gcv2
Gdh1
Glt1
Gln1
His1
His4
His7
Hom2
Hom6
Ilv2
Ilv5
Ilv3
Met5
Ser1
Ser3
Ser2
Trp1
Trp5
SUC
FUM
Frd1
Fum1
Lsc1
Uga2
SucCOA
MAL
Mdh1
Mdh2
Mdh3
Kgd1
Kgd2
Mls1
Icl1
Idp1
AKG
OXA
Pyc1
Pyc2
Pck1
Idh1
Idh2
Cit1
ISOCIT
ACO
CIT
AcCOA
PYR
ACALD
ACETATE
ETOH
Adh1
Adh2
Adh3
Adh6
AcCOA
Acs2
Ach1
Aco1
Aco2
Pdb1
Pda1
Lat1
Pdc1
Pdc5
Pdc6
Ald4
Ald5
Ald6
< 0.3
1.0
> 3.0
Relative expression to wild type

tdh3Δ

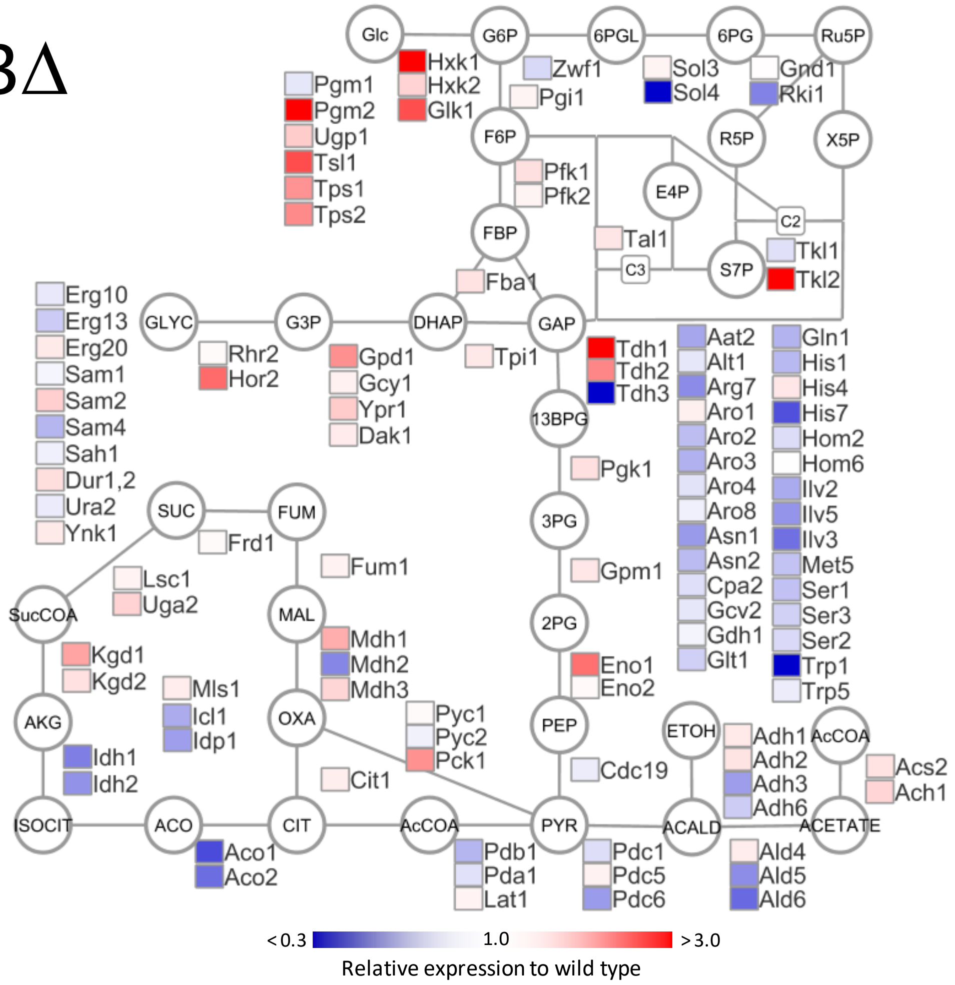

Glc
G6P
6PGL
6PG
Ru5P
Hxk1
Hxk2
Glk1
Zwf1
Pgi1
Sol3
Sol4
Gnd1
Rki1
Pgm1
Pgm2
Ugp1
Tsl1
Tps1
Tps2
F6P
R5P
X5P
Pfk1
Pfk2
E4P
C2
FBP
Tal1
C3
S7P
Tkl1
Tkl2
Fba1
Erg10
Erg13
Erg20
Sam1
Sam2
Sam4
Sah1
Dur1,2
Ura2
Ynk1
GLYC
G3P
DHAP
GAP
Rhr2
Hor2
Gpd1
Gcy1
Ypr1
Dak1
Tpi1
Tdh1
Tdh2
Tdh3
13BPG
Pgk1
3PG
Gpm1
2PG
Eno1
Eno2
PEP
Cdc19
Aat2
Alt1
Arg7
Aro1
Aro2
Aro3
Aro4
Aro8
Asn1
Asn2
Cpa2
Gcv2
Gdh1
Glt1
Gln1
His1
His4
His7
Hom2
Hom6
Ilv2
Ilv5
Ilv3
Met5
Ser1
Ser3
Ser2
Trp1
Trp5
SUC
FUM
Frd1
Fum1
Lsc1
Uga2
SucCOA
MAL
Mdh1
Mdh2
Mdh3
Kgd1
Kgd2
Mls1
Icl1
Idp1
AKG
OXA
Pyc1
Pyc2
Pck1
Idh1
Idh2
Cit1
ISOCIT
ACO
CIT
AcCOA
PYR
ETOH
Adh1
Adh2
Adh3
Adh6
AcCOA
Acs2
Ach1
ACALD
ACETATE
Aco1
Aco2
Pdb1
Pda1
Lat1
Pdc1
Pdc5
Pdc6
Ald4
Ald5
Ald6
< 0.3
1.0
> 3.0
Relative expression to wild type

tkl1Δ

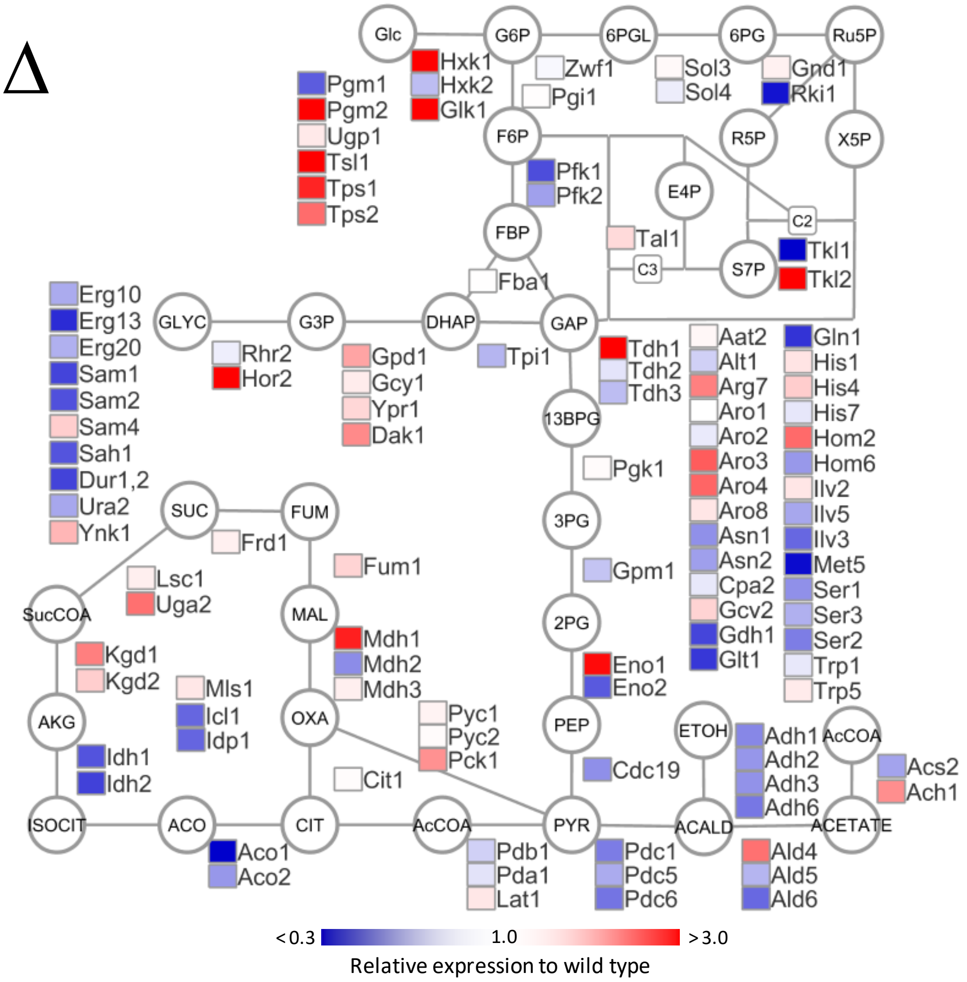
Glc
G6P
6PGL
6PG
Ru5P
Hxk1
Hxk2
Glk1
Zwf1
Pgi1
Sol3
Sol4
Gnd1
Rki1
Pgm1
Pgm2
Ugp1
Tsl1
Tps1
Tps2
F6P
R5P
X5P
Pfk1
Pfk2
E4P
C2
FBP
Tal1
Tkl1
Tkl2
C3
S7P
Fba1
Erg10
Erg13
Erg20
Sam1
Sam2
Sam4
Sah1
Dur1,2
Ura2
Ynk1
GLYC
G3P
DHAP
GAP
Rhr2
Hor2
Gpd1
Gcy1
Ypr1
Dak1
Tpi1
Tdh1
Tdh2
Tdh3
13BPG
Pgk1
3PG
Gpm1
2PG
Eno1
Eno2
PEP
Cdc19
PYR
Aat2
Alt1
Arg7
Aro1
Aro2
Aro3
Aro4
Aro8
Asn1
Asn2
Cpa2
Gcv2
Gdh1
Glt1
Gln1
His1
His4
His7
Hom2
Hom6
Ilv2
Ilv5
Ilv3
Met5
Ser1
Ser3
Ser2
Trp1
Trp5
SUC
FUM
Frd1
Fum1
SucCOA
Lsc1
Uga2
MAL
Mdh1
Mdh2
Mdh3
Kgd1
Kgd2
Mls1
Icl1
Idp1
AKG
OXA
Pyc1
Pyc2
Pck1
Idh1
Idh2
Cit1
ISOCIT
ACO
CIT
AcCOA
Aco1
Aco2
Pdb1
Pda1
Lat1
Pdc1
Pdc5
Pdc6
ETOH
Adh1
Adh2
Adh3
Adh6
AcCOA
Acs2
Ach1
ACALD
ACETATE
Ald4
Ald5
Ald6
< 0.3
1.0
> 3.0
Relative expression to wild type

tps2Δ

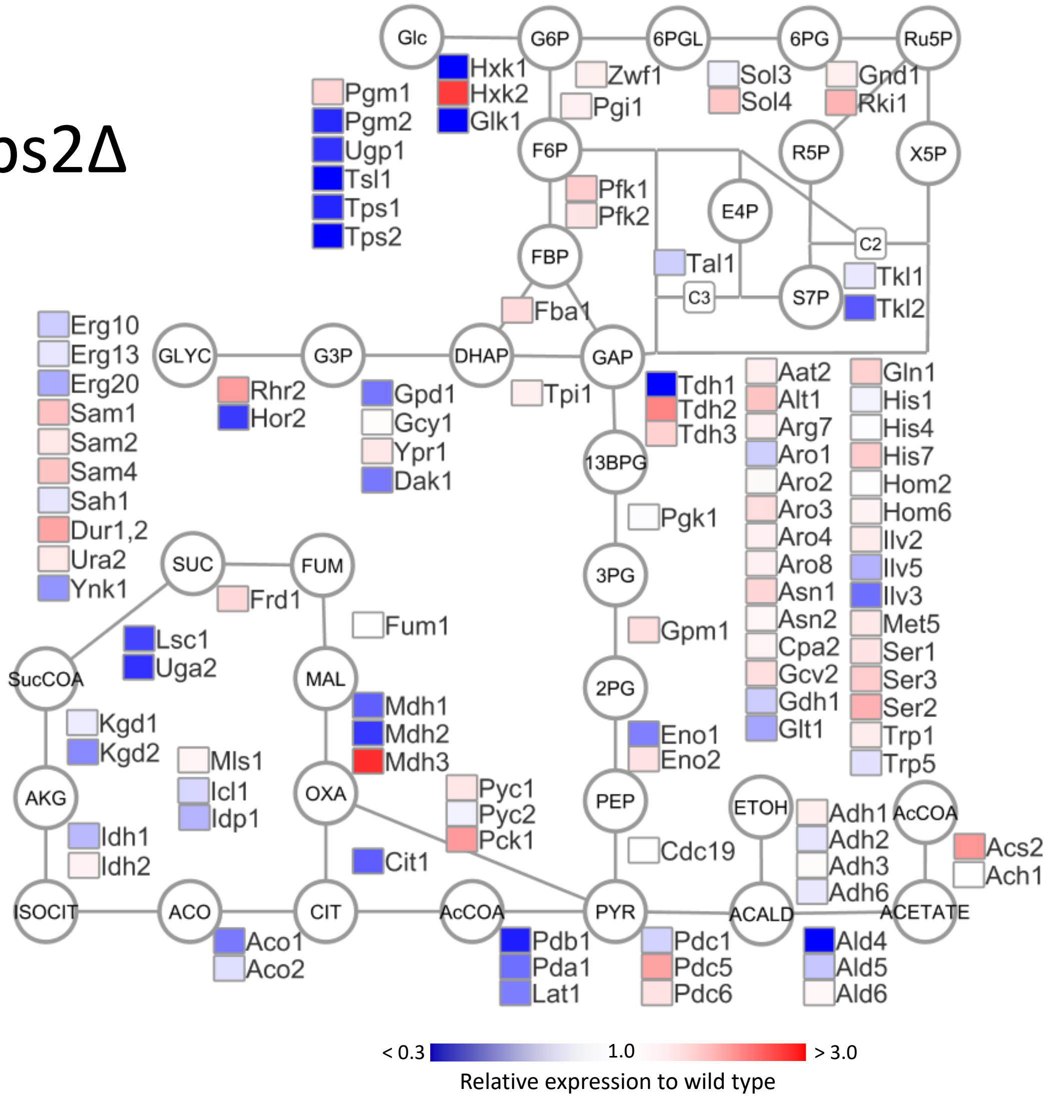

Glc
G6P
6PGL
6PG
Ru5P
Hxk1
Hxk2
Glk1
Zwf1
Pgi1
Sol3
Sol4
Gnd1
Rki1
Pgm1
Pgm2
Ugp1
Tsl1
Tps1
Tps2
F6P
R5P
X5P
Pfk1
Pfk2
E4P
C2
FBP
Tal1
Tkl1
Tkl2
C3
S7P
Fba1
Erg10
Erg13
Erg20
Sam1
Sam2
Sam4
Sah1
Dur1,2
Ura2
Ynk1
GLYC
G3P
DHAP
GAP
Rhr2
Hor2
Gpd1
Gcy1
Ypr1
Dak1
Tpi1
Tdh1
Tdh2
Tdh3
13BPG
Pgk1
3PG
Gpm1
2PG
Eno1
Eno2
PEP
Cdc19
Aat2
Alt1
Arg7
Aro1
Aro2
Aro3
Aro4
Aro8
Asn1
Asn2
Cpa2
Gcv2
Gdh1
Glt1
Gln1
His1
His4
His7
Hom2
Hom6
Ilv2
Ilv5
Ilv3
Met5
Ser1
Ser3
Ser2
Trp1
Trp5
SUC
FUM
Frd1
Fum1
Lsc1
Uga2
SucCOA
MAL
Mdh1
Mdh2
Mdh3
Kgd1
Kgd2
Mls1
Icl1
Idp1
AKG
OXA
Pyc1
Pyc2
Pck1
Idh1
Idh2
Cit1
ETOH
Adh1
Adh2
Adh3
Adh6
AcCOA
Acs2
Ach1
ISOCIT
ACO
CIT
AcCOA
PYR
ACALD
ACETATE
Aco1
Aco2
Pdb1
Pda1
Lat1
Pdc1
Pdc5
Pdc6
Ald4
Ald5
Ald6
< 0.3
1.0
> 3.0
Relative expression to wild type

zwf1Δ

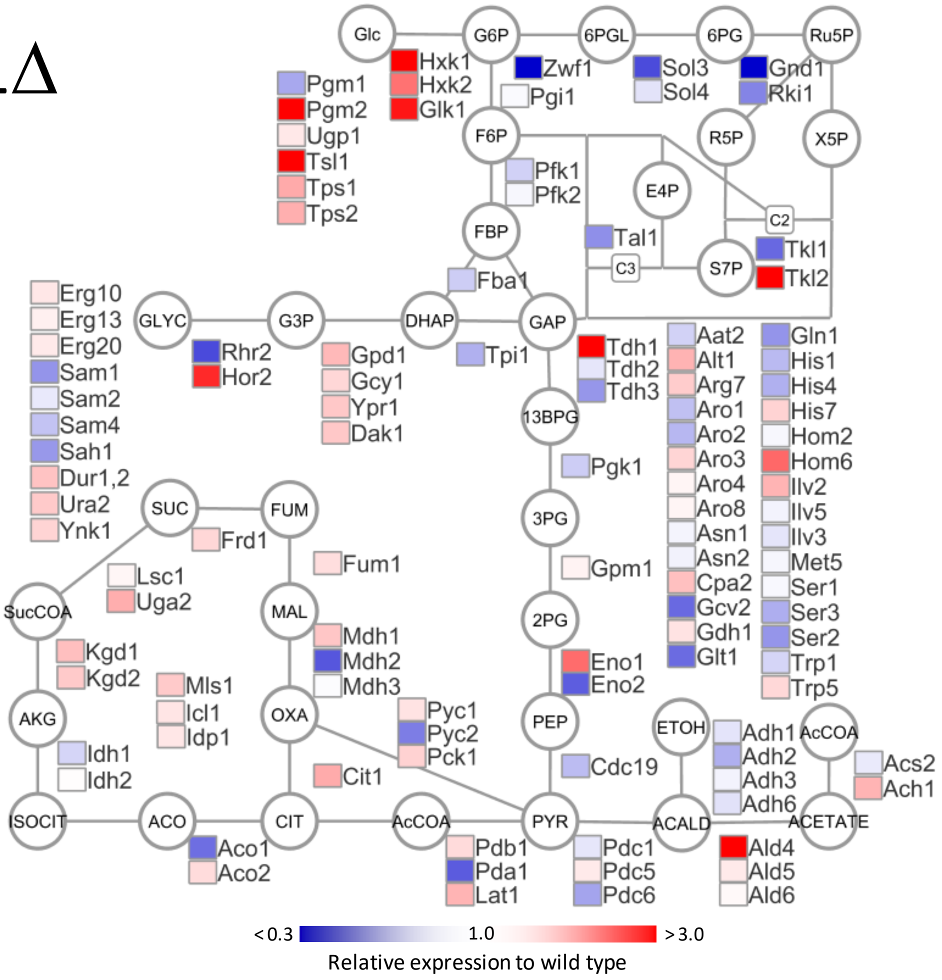

Glc
G6P
6PGL
6PG
Ru5P
Hxk1
Hxk2
Glk1
Zwf1
Pgi1
Sol3
Sol4
Gnd1
Rki1
Pgm1
Pgm2
Ugp1
Tsl1
Tps1
Tps2
F6P
R5P
X5P
Pfk1
Pfk2
E4P
C2
FBP
Tal1
C3
S7P
Tkl1
Tkl2
Fba1
Erg10
Erg13
Erg20
Sam1
Sam2
Sam4
Sah1
Dur1,2
Ura2
Ynk1
GLYC
G3P
DHAP
GAP
Rhr2
Hor2
Gpd1
Gcy1
Ypr1
Dak1
Tpi1
Tdh1
Tdh2
Tdh3
13BPG
Pgk1
3PG
Gpm1
2PG
Eno1
Eno2
PEP
Cdc19
PYR
Aat2
Alt1
Arg7
Aro1
Aro2
Aro3
Aro4
Aro8
Asn1
Asn2
Cpa2
Gcv2
Gdh1
Glt1
Gln1
His1
His4
His7
Hom2
Hom6
Ilv2
Ilv5
Ilv3
Met5
Ser1
Ser3
Ser2
Trp1
Trp5
SUC
FUM
Frd1
Fum1
Lsc1
Uga2
SucCOA
MAL
Mdh1
Mdh2
Mdh3
Kgd1
Kgd2
AKG
Mls1
Icl1
Idp1
OXA
Pyc1
Pyc2
Pck1
Idh1
Idh2
Cit1
ISOCIT
ACO
CIT
AcCOA
Aco1
Aco2
Pdb1
Pda1
Lat1
Pdc1
Pdc5
Pdc6
ETOH
Adh1
Adh2
Adh3
Adh6
AcCOA
Acs2
Ach1
ACALD
ACETATE
Ald4
Ald5
Ald6
< 0.3
1.0
> 3.0
Relative expression to wild type
