# Supplementary material for: Targeted proteome analysis of single-gene deletion strains of Saccharomyces cerevisiae lacking enzymes in the central carbon metabolism
Source: PLoS One. 2017 Feb 27;12(2):e0172742. doi: 10.1371/journal.pone.0172742 (PMC5328394; doi:10.1371/journal.pone.0172742)

S2 Fig. Protein investment for the biosynthesis of 110 enzymes. (a) Copy numbers of 110 enzymes per cell. (b) Total numbers of amino acids required for 110 enzymes per cell. Data were calculated from the dataset by Kulak *et al*, the targeted proteome data obtained in this study, and amino acid sequence of each enzyme. Data are shown as the mean of triplicate analysis.

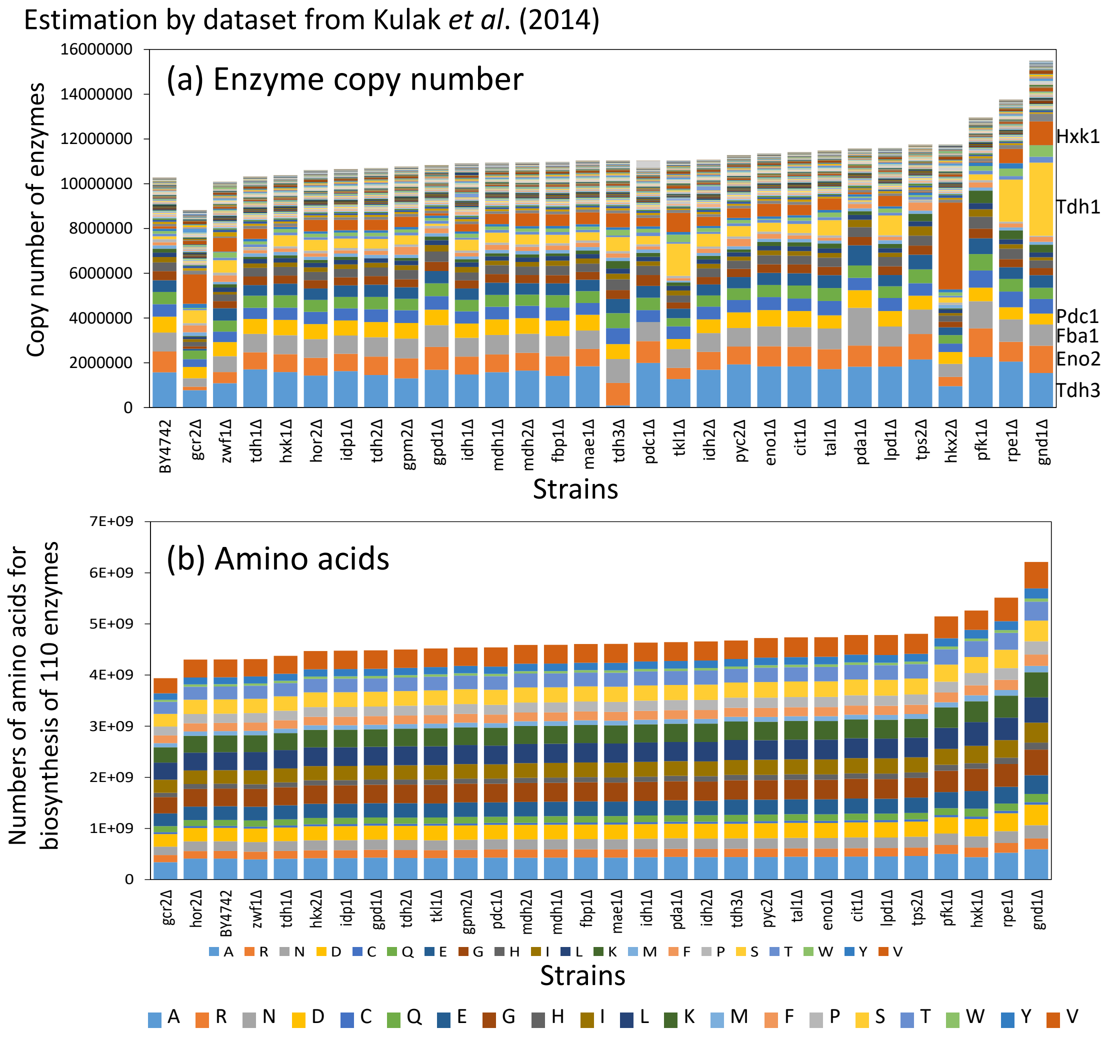

Supplement: S2 Fig — (PDF) [file pone.0172742.s002.pdf]
