# Supplementary material for: Targeted proteome analysis of single-gene deletion strains of Saccharomyces cerevisiae lacking enzymes in the central carbon metabolism
Source: PLoS One. 2017 Feb 27;12(2):e0172742. doi: 10.1371/journal.pone.0172742 (PMC5328394; doi:10.1371/journal.pone.0172742)

S3 Fig. Copy numbers of 110 enzymes per cell. Copy numbers of 110 enzymes in each mutant strain were calculated from the dataset by (a) Ghaemmanhami *et al.* (2003) and (b) by de-Gody *et al.* (2008). Data are shown as the mean of triplicate analysis.

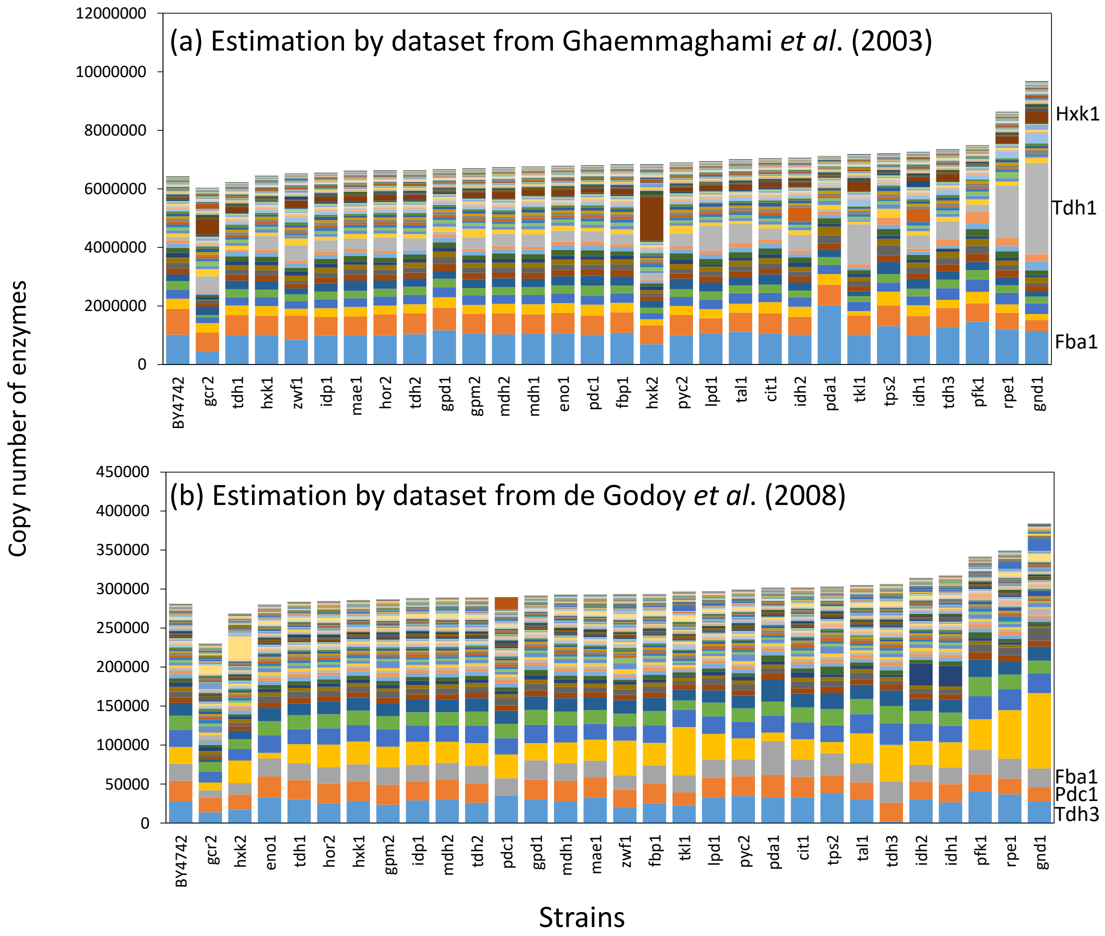

Supplement: S3 Fig — (PDF) [file pone.0172742.s003.pdf]
