# Supplementary material for: Targeted proteome analysis of single-gene deletion strains of Saccharomyces cerevisiae lacking enzymes in the central carbon metabolism
Source: PLoS One. 2017 Feb 27;12(2):e0172742. doi: 10.1371/journal.pone.0172742 (PMC5328394; doi:10.1371/journal.pone.0172742)

S4 Fig

Distributions of Spearman's rank-order correlation coefficients across all enzymes (All data, n = 5995 enzyme pairs). Null distribution obtained from the randomized data was also represented.

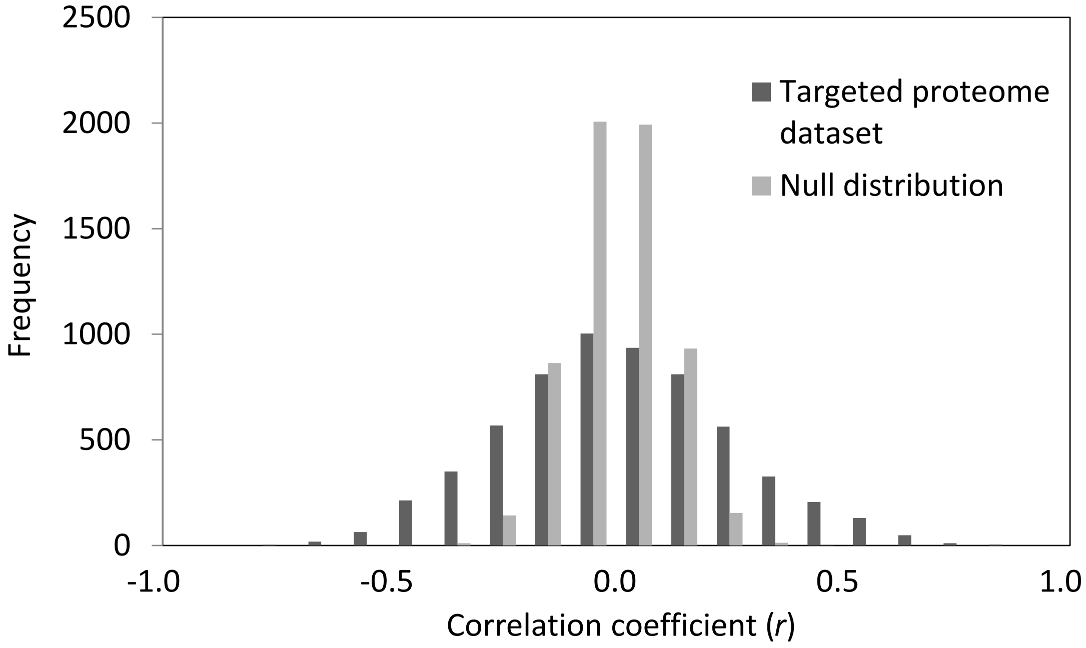

Supplement: S4 Fig — (PDF) [file pone.0172742.s004.pdf]
